# RNA polymerase I inhibition induces terminal differentiation, growth arrest, and vulnerability to senolytics in colorectal cancer cells

**DOI:** 10.1002/1878-0261.13265

**Published:** 2022-07-01

**Authors:** Christoph Otto, Carolin Kastner, Stefanie Schmidt, Konstantin Uttinger, Apoorva Baluapuri, Sarah Denk, Mathias T. Rosenfeldt, Andreas Rosenwald, Florian Roehrig, Carsten P. Ade, Christina Schuelein‐Voelk, Markus E. Diefenbacher, Christoph‐Thomas Germer, Elmar Wolf, Martin Eilers, Armin Wiegering

**Affiliations:** ^1^ Experimental Visceral Surgery, Department of General, Visceral, Transplantation, Vascular and Pediatric Surgery (Department of Surgery I) University Hospital Würzburg Germany; ^2^ Department of Biochemistry and Molecular Biology, Biocenter University of Würzburg Germany; ^3^ Department of General, Visceral, Transplantation, Vascular and Pediatric Surgery (Department of Surgery I) University Hospital Würzburg Germany; ^4^ Institute of Pathology Universität of Würzburg Germany; ^5^ Comprehensive Cancer Center Mainfranken University of Würzburg Germany

**Keywords:** CRC, CX5461, MIZ1, MYC, ribosome, RNAPOL1

## Abstract

Ribosomal biogenesis and protein synthesis are deregulated in most cancers, suggesting that interfering with translation machinery may hold significant therapeutic potential. Here, we show that loss of the tumor suppressor adenomatous polyposis coli (APC), which constitutes the initiating event in the adenoma carcinoma sequence for colorectal cancer (CRC), induces the expression of RNA polymerase I (RNAPOL1) transcription machinery, and subsequently upregulates ribosomal DNA (rDNA) transcription. Targeting RNAPOL1 with a specific inhibitor, CX5461, disrupts nucleolar integrity, and induces a disbalance of ribosomal proteins. Surprisingly, CX5461‐induced growth arrest is irreversible and exhibits features of senescence and terminal differentiation. Mechanistically, CX5461 promotes differentiation in an MYC‐interacting zinc‐finger protein 1 (MIZ1)‐ and retinoblastoma protein (Rb)‐dependent manner. In addition, the inhibition of RNAPOL1 renders CRC cells vulnerable towards senolytic agents. We validated this therapeutic effect of CX5461 in murine‐ and patient‐derived organoids, and in a xenograft mouse model. These results show that targeting ribosomal biogenesis together with targeting the consecutive, senescent phenotype using approved drugs is a new therapeutic approach, which can rapidly be transferred from bench to bedside.

## Graphical abstract

Loss of APC, the initiating event in the adenoma carcinoma sequence for CRC, induces the expression of RNAPOL1, and subsequently upregulates rDNA transcription. Targeting RNAPOL1 with a specific inhibitor, CX5461, disrupts nucleolar integrity, induces imbalance of ribosomal proteins, irreversible growth arrest with features of senescence and terminal differentiation. This inhibition of RNAPOL1 renders CRC cells vulnerable towards senolytic agents.

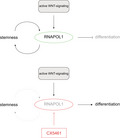

**Abbreviations**
APC: adenomatous polyposis coli
CRC: colorectal cancer
GSEA: gene set enrichment analysis
Hb: hemoglobin
MTO: murine tumor organoids
MIZ1: MYC‐interacting zinc‐finger protein 1
NPM1: nucleophosmin
TT: primary CRC samples
Rb: retinoblastoma protein
rDNA: ribosomal DNA
POLR1A: RNA polymerase I subunit A
RNAPOL1: RNA polymerase I
NT: untransformed mucosa
WT: wild‐type

## 1 Introduction

Colorectal cancer (CRC) is one of the most frequent malignancies in industrial nations accounting for more than 1 million deaths worldwide per year [1]. There is an increasing number of reports showing that tumors exhibit varying dependencies for translation factors and ribosomal proteins opening potential therapeutic windows [2, 3, 4]. This also can be found in CRC [5, 6, 7].

Activation of the WNT signaling pathway—either by bi‐allelic loss or mutation of the tumor suppressor gene APC or activating mutations within beta‐catenin—is the molecular hallmark in virtually all CRCs [8]. One of the key features of activated WNT signaling is an upregulation of the MYC protooncogene, which drives DNA transcription via RNA polymerase II (RNAPOLII) [9]. In addition, MYC can accelerate the transcription of genes encoding translation initiation factors, ribosomal proteins, and ribosomal RNA (rRNA) [6, 10, 11]. In line with this, in the majority of cancers, an upregulation of rDNA transcription is found and the level of elevated rRNA expression correlates with patient prognosis [12, 13, 14].

Recent evidence shows that in CRC the ability of protein translation is limited to a subset of tumor cells, which reside adjacent to the tumor stroma, while cells within the tumor center lose the capacity for active protein synthesis and show a more differentiated phenotype [15]. Cancer cells with active protein translation are characterized by elevated levels of RNA Polymerase I subunit A (POLR1A), one of the key components of RNAPOL1. Depletion of POLR1A either by genetic approach or by using a degrader induces an irreversible growth arrest. Furthermore, high levels of POLR1A define a stemness compartment independent of LGR5 expression and drive *in vivo* tumor formation of LGR5 low cells. In addition, depletion of POLR1A in LGR5 high cells prevents tumor formation [15].

This impact of POLR1A translates into a therapeutic opportunity as RNAPOL1 activity is conversely required to maintain stemness in CRC. The small molecule CX5461 is a potent and selective inhibitor of RNAPOL1 function. Promising therapeutic effects have been shown for different malignancies including lymphoma and hematological cancer, and it is currently in phase I clinical trials [16, 17, 18].

By using human tumor samples, murine, and patient‐derived organoids, we show that RNAPOL1‐dependent rDNA transcription is strictly dependent on active WNT signaling. Consequently, inhibition of RNAPOL1 transcriptional activity by CX5461 disrupts ribosomal protein distribution and nucleolar formation leading to an irreversible growth arrest and to an induction of features of terminal differentiation and senescence in CRC cells *in vitro* and *in vivo*. This phenotype opens a therapeutic window for senolytic drugs to have an additional effect on CRC and possibly also other cancers.

## 2 Material and methods

### 2.1 Cell culture

DLD1, HT29, LS174T, and SW480 cells were cultured in RPMI 1640 (Thermo Fisher Scientific, 21 875 091, Dreieich, Germany), whereas HCT116 p53^+/+^, HCT116 p53^−/−^, and HEK293T cells were cultured in DMEM (Thermo Fisher Scientific, 41 966 052). All media was supplemented with 10% (v/v) heat‐inactivated fetal calf serum (FCS, Pan Biotech, P30‐2302, Aidenbach, Germany), 1% penicillin–streptomycin (Sigma, P4333, Taufkirchen, Germany). All cell lines were purchased from American Type Culture Collection. L17 R‐spondin‐producing cells were maintained in DMEM with 1 mg·mL^−1^ G418 (Sigma, 4 727 878 001). L17 R‐spondin cells were a gift from O. Sansom (Beatson Institute, Glasgow). The cultures were maintained in a humidified atmosphere with 5% CO_2_ at 37 °C (standard incubator conditions). Cells were validated by short‐tandem repeat genotyping (https://clsgmbh.de) prior to starting experiments. A test for mycoplasma contamination was performed regularly (Minerva Biolabs GmbH, Berlin, Germany). Cells were used for less than 20 passages after revitalization.

Where specified, the following reagents were added: doxycycline (Sigma, D9891), CX‐5461 (Sellekchem, S2684, München, Germany), Ouabain (Sigma,11 018–89‐6), Navitoclax (Sellekchem, S1001), THZ1 (Sellekchem, S7549).

### 2.2 Organoid culture

Isolation and culture of patient‐derived organoids were approved by the ethics committee of the University of Würzburg (#142/16‐ge). The study methodologies conformed to the standards set by the Declaration of Helsinki. The experiments were undertaken with the understanding and written consent of each subject. Isolation and culture of murine, and human patient‐derived organoids were performed as described elsewhere [19].

### 2.3 Lentiviral transduction and transfection

All shRNA experiments were carried out by stable lentiviral transduction. Lentiviruses were generated by transfection of HEK293T cells with pInducer10 (Trono Laboratory, N/A, Lausanne, Switzerland) or pGIPZ (Dharmacon, N/A, Schwerte, Germany) together with the packaging plasmids psPAX.2 (Trono Laboratory, Addgene 12 260) and pMD.2G (Trono Laboratory, Addgene 12259). shRNA sequences are listed in Table S1. shRNAs against Miz1 were selected as described by Fellmann. Infections were carried out using 8 μg·mL^−1^ polybrene (107 689, Sigma). Forty‐eight hours after infection, cells were selected with 2 μg·mL^−1^ puromycin (ant‐pr, Invivogen, Toulouse, France), and pools of selected cells were used for downstream analyses.

### 2.4 Cell viability assay and determination of IC50 values

Exponentially growing cells (5 × 103 cells/well in 200 μL of culture medium) were cultured in 96‐well flat‐bottom tissue plates (Greiner Bio‐One, Frickenhausen, Germany). The next day, the culture medium was replaced, and the cells were treated with CX‐5461 at concentrations from 10^−8^ to 10^−5^ m for 72 h under standard incubator conditions. Cell viability was determined by WST‐8 assay according to the manufacturer's instructions (PromoCell GmbH, Heidelberg, Germany). Absorbance was measured at 450 nm with a microplate reader. WST‐8 is reduced by cellular dehydrogenases to an orange formazan product that is soluble in the culture medium. The amount of formazan produced is directly proportional to the number of living cells. Based on absorbance values, the IC50 values (half‐maximal inhibitory concentration) and their 95% confidence interval were calculated with nonlinear regression fit to a sigmoidal dose–response curve using prism 5 statistical software (GraphPad Software, Inc., San Diego, CA, USA).

### 2.5 Colony formation assay

Cells (0.5–1.0 × 10^5^) were seeded on six‐well plates, fixed at the end of treatment with 4% formaldehyde, and stained with 0.25% crystal violet in 20% methanol. The culture plates were imaged using EVOS Cell Imaging System (ThermoFisher Scientific).

### 2.6 Flow cytometry analysis

For FACS using propidium iodide (Sigma, 81 845), cells were collected by trypsinization, washed with cold PBS, and fixed in 80% ethanol overnight at −20 °C. After washing with PBS, the cells were resuspended in PBS with RNase A (24 μg·mL^−1^) and propidium iodide (54 μm), and incubated for 30 min at 37 °C.

For FACS using annexin V and propidium iodide, the supernatant of the respective cultures was combined with cells collected by trypsinization and washed with cold PBS. Cell pellets were resuspended in 100 μL 1× annexin V‐binding buffer (10 mm HEPES pH 7.4, 140 mm NaCl, and 2.5 mm CaCl_2_) and 2 μL annexin V/Pacific Blue dye and incubated for 15 min at room temperature in the dark. Afterwards, 400 μL 1× binding buffer and propidium iodide (54 μm) were added, and the samples were stored cold and dark until analysis.

Subsequent analysis of all FACS experiments was performed on a BD FACSCanto II flow cytometer using bd facsdiva Software, Bio Science, Heidelberg,Germany).

### 2.7 Growth curve

1.8 × 10^3^ cells were seeded on a 96 well plate. Initial measurement as timepoint zero was done 16 h after seeding. Inhibitor/control vehicle treatment was started immediately before timepoint zero measurement. Seventy‐two hours medium was changed to inhibitor/control vehicle free medium after washing two times with PBS. Measurement was done with Operetta High Content Imaging System with 20× magnification in Brightfield mode every 24 h. Images were processed using harmony high content imaging and analysis Software (Keyence Deutschland GmbH, Neu‐Isenburg, Germany).

### 2.8 Immunofluorescence staining and high content imaging

For high content imaging, cells and organoids were cultivated in 96‐well plates (Greiner Bio‐One), for confocal microscopy cells were plated on coverslips. In the case of EdU pulse labelling, cells and organoids were pulsed with 5 μm EdU (Thermo Fisher Scientific) for 25 min immediately before fixation. Afterwards they were fixed in freshly diluted 3.7% paraformaldehyde in PBS for 10 min (cells) or 15 min (organoids). After washing with PBS, cells and organoids were permeabilized with 0.2% Triton X‐100/PBS for 10 min (cells) or 25 min (organoids) and blocked in 5% BSA/PBS for at least 1 h. In the case of EdU pulse labelling, after washing with PBS, EdU Click reaction was carried out according to the manufacturer's protocol (Click‐iT™ EdU Cell Proliferation Kit for Imaging, Thermo Fisher Scientific). Samples were stained with primary antibodies diluted in 5% BSA in PBS overnight at 4 °C and after rinsing with PBS, incubated with the corresponding fluorescence‐labeled secondary antibody for 1 h at room temperature. Nuclear counterstaining was performed using Hoechst 33342 (Sigma‐Aldrich, B2261) for 5 min or samples were mounted with ProLong Gold Antifade Reagent with DAPI (Cell Signaling Technology, Leiden, the Netherlands). For high content imaging, images were taken with an Operetta High Content Imaging System with 20× or 40× magnification. Images were processed using harmony high content imaging and analysis Software, r and prism9. In other cases, images were taken with a confocal laser scanning microscope (Zeiss LSM 780). Primary and secondary antibodies are listed in Table S2.

### 2.9 Immunohistochemistry

Immunohistochemistry was performed on 5‐μm paraffin sections according to standard procedures. Sections were deparaffinized with xylene and rinsed in decreasing concentrations of ethanol prior to unmasking by heating for 5 min with 10 mm sodium citrate buffer in a microwave oven at 600 W. Endogenous peroxidase was quenched with 3% hydrogen peroxide in methanol for 10 min. Sections were washed with PBS, blocked for 15 min in 1% goat serum, and incubated with primary antibodies overnight at 4 °C diluted in antibody diluent buffer (DAKO). Sections were washed and incubated with the corresponding HRP‐conjugated secondary antibody diluted in antibody diluent buffer (DAKOy) for 1 h. After development in 5% 3,3′‐diaminobenzidine (DAKO), and counterstaining with haematoxilin, the sections were dehydrated in graded ethanol and embedded *in Vitro* Clud (Langenbrinck). Antibodies used are listed in Table S2.

### 2.10 Immunoblot

Cells were lysed in pre‐cooled RIPA buffer (50 mm Tris buffer pH 7.5, 150 mm NaCl, 1% NP‐40, 0.5% sodium deoxycholate, 0.1% SDS; Sigma, P8340) and phosphatase inhibitors (Sigma, P5726 and P0044), and incubated for 30 min on ice with intermittent vortexing. The lysate was cleared by centrifugation, and protein concentration was determined using the BCA assay. Equal amounts of proteins were loaded on a polyacrylamide gel (SDS/PAGE), electrophoresed, and transferred to a PVDF membrane (Millipore, Darmstadt, Germany). Membranes were blocked for 1 h and probed using indicated primary antibodies in 5% BSA in PBS overnight at 4 °C. For visualization, the LAS4000 Imaging System was used (fuji). All antibodies are listed in Table S2.

### 2.11 Organoid viability assay

For analysis of the viability of human and murine organoids, organoids were mechanically disrupted and centrifuged at 700 ***g*** for 4 min. The supernatant was removed, and the organoid pellet was resuspended in the desired amount of Matrigel thoroughly. The same volume of organoid‐containing gel matrix was plated into wells of prewarmed 96‐well or 48‐well plate (Cellstar®, Greiner Bio‐one). CX‐5461 treatment was started 24–48 h after seeding, depending on the type of organoid and therefore the time necessary to set up small established organoids after the mechanical disruption. Desired concentration of inhibitor was added to the culture medium. After 96 h of treatment CellTiter‐Blue® solution (Promega, G8080, Walldorf, Germany) was added (10 μL per 50 μL medium—according to supplier's recommendation). After 4.5 h measurement was done using Mikroplate reader (Tecan, Spark, Männedorf, Switzerland).

### 2.12 Protein synthesis rates quantification

Protein synthesis rates were quantified using the Click‐iT Plus OPP Alexa Fluor 488 Protein Synthesis Assay Kit according to the manufacturer's instructions (Thermo Fisher Scientific). Cells were seeded on 12‐well plates (ibidi) and treated with 20 mm OPP for 30 min under normal culture conditions. The OPP accumulation was stopped by fixing the cells with 4.0% formaldehyde in PBS for 15 min at room temperature followed by permeabilization with 0.5% Triton X‐100 in PBS for 15 min at room temperature. Cells were mounted with ProLong Gold Antifade Reagent with DAPI (Cell Signaling Technology, 8961).

### 2.13 Quantitative PCR (qPCR)

Total RNA was extracted from 10^6^ cells with Trizol (Invitrogen Life Technologies), quantified (Nanodrop ND‐1000, Thermo Fisher Scientific) and assessed for RNA integrity (Experion, Bio‐Rad Laboratories, Feldkirchen, Germany). RNA (1 μg) was reverse transcribed using M‐MLV reverse transcriptase (Promega, M1701; 25 °C for 5 min, 37 °C for 60 min, 85 °C for 5 min). qPCR was performed with PowerUP SYBR Green qPCR MasterMix (Thermo Fisher Scientific). The cycler protocol was 2 min at 50 °C, 2 min at 95 °C, 40 cycles of 15 s at 95 °C, 60 s at 60 °C, and 5 min at 72 °C. Postamplification melting curves were controlled to exclude primer‐dimer artifacts and contaminations. The reference genes *PPIA* (peptidylprolyl isomerase A), *ACTB* (β‐actin), *B2M* (ß2 Mikroglobulin), *GAPDH* (Glycerinaldehyde‐3‐phosphate‐dehydrogenase) and *RPLO* (Ribosomal Protein Lateral Stalk Subunit P0) were used for normalization, and mRNA and rRNA expression levels were calculated with the ∆∆Cq method. Primer sequences are listed in Table S3.

### 2.14 RNA sequencing

For global gene expression analysis, total RNA was isolated from cells using the RNeasy Mini Kit (Qiagen, 74 106, Stockach, Germany) with on‐column DNase I digestion according to the instruction manual. mRNA was isolated using NEBNext Poly(A) mRNA Magnetic Isolation Module (NEB, Frankfurt am Main, Germany), and library preparation was performed with the NEBNext Ultra RNA Library Prep Kit for Illumina following the manufacturer's instructions. Libraries were size‐selected using Agentcourt AMPure XP Beads (Beckman Coulter), followed by amplification with 12 PCR cycles. RNA and final library quality, and concentration, were assessed on the Fragment Analyzer (Agilent, Santa Clara, CA, USA) by using Standard Sensitivity RNA Analysis Kit (Agilent). Libraries were sequenced on an Illumina Genome Analyzer IIx following the manufacturer's instructions.

For RNA‐sequencing analysis, reads were aligned to the human genome (hg19) with bowtie v.0.12.8 (Wolters Kluwer, Alphen aan den Rijn, the Netherlands) using default parameters. Mapped reads per gene (Ensembl GRCh37, release 74) were counted using the ‘summarizeOverlaps’ function in the GenomicAlignments R package. Nonexpressed genes were removed (mean read count per gene over all samples of > 1) and trimmed mean of *M*‐values normalization was performed using edger (https://bioconductor.org). GSEA was performed using the C2, C5, and Hallmark collections from the MSigDB v.6.0 with default parameters and 1000 permutations.

Original data and analyses are available at GSE with the numbers GSE204749 for RNA‐Seq of LS174t cells with CX5461 and GSE106858 for 2 APC(del)/APC(res) cells from [5].

### 2.15 SA‐β‐galactosidase staining

SA‐β‐gal staining was performed as former described: Cells were fixed in PBS buffer containing 4% (w/v) formaldehyde and 0.25% (w/v) glutaraldehyde for 15 min followed by three washes with PBS/MgCl_2_ (1 mm, pH 6.0). Next, cells were incubated in phosphate buffer pH 6.0 containing 1 mg·mL^−1^ 5‐bromo‐4‐chloro‐3‐indolyl‐b‐d‐galactopyranoside, 40 mm citric acid/sodium phosphate (pH 6.0), 5 mm potassium ferrocyanide, 5 mm potassium ferricyanide, 150 mm NaCl, and 2 mm MgCl_2_ for 17 h at 37 °C. After incubation, cells were washed three times in PBS followed by ethanol (70%, v/v) treatment. The culture plates were imaged using EVOS Cell Imaging System (ThermoFisher Scientific).

### 2.16 CRC xenograft studies

All animal studies were authorized by the local ethics committee (Government of Lower Franconia, Germany, animal license number 55.2–2532–2‐405), and mice were treated according to institutional and European Union guidelines. Female NMRI Nude (Rj:NMRI‐Foxn1nu/nu) mice at 10 weeks of age were purchased from Janvier Labs (Saint Berthevin, France) and were housed in groups of five animals per cage (Makrolon type III) with free access to sterile food and water. The cages were placed in ventilated filter cabinets with humidity and temperature control (Scanbur, Karlslunde, Denmark) within the specified pathogen‐free animal laboratory facility with a temperature of 22 °C, a humidity of 45%, and a 12 h light/12 h dark cycle.

To generate CRC xenografts, 2.0 × 10^6^ HCT116 cells resuspended in 200 μL phosphate‐buffered saline were injected subcutaneously into the left hind flank of athymic female NMRI‐Foxn1^nu^ (nude) mice aged 8–10 weeks with 24–27 g body weight (Janvier, Le Genest‐Saint‐Isle, France). Tumors were measured using calipers and tumor volume (in mm^3^) was calculated using the ellipsoid formula A2 × B × π/6, where A represents the smaller diameter [20]. When tumors reached a volume of ~ 50 mm^3^, mice were randomized into the vehicle (a mixture of DMSO (5%), cyclodextrin (5%), 1,2‐propanediol (10%) and dextrose (10%)) or CX‐5461 (Selleckchem) treatment groups. Mice in therapy groups were treated daily with 50 mg·kg^−1^ CX‐5461 dissolved in the vehicle by oral gavage until tumors reached tumor volume of ~ 600 mm^3^ (the endpoint of experiment). Ouabain (1 mg·kg^−1^ in PBS, Sigma) was administrated intravenously once daily three times weekly (Mo, Mi, Fr treatment with weekend rest). The tumor size and body weight of all animals were measured every day. All animal experiments were authorized by the local ethics committee, and mice were treated according to institutional and European Union guidelines.

### 2.17 Statistical analysis

Experiments were performed at least three times with replicate samples. Data are plotted as means ± SD (standard deviation). The means were compared using analysis of variance (ANOVA) plus Bonferroni's *t*‐test.

## 3 Results

**Fig. 1:**
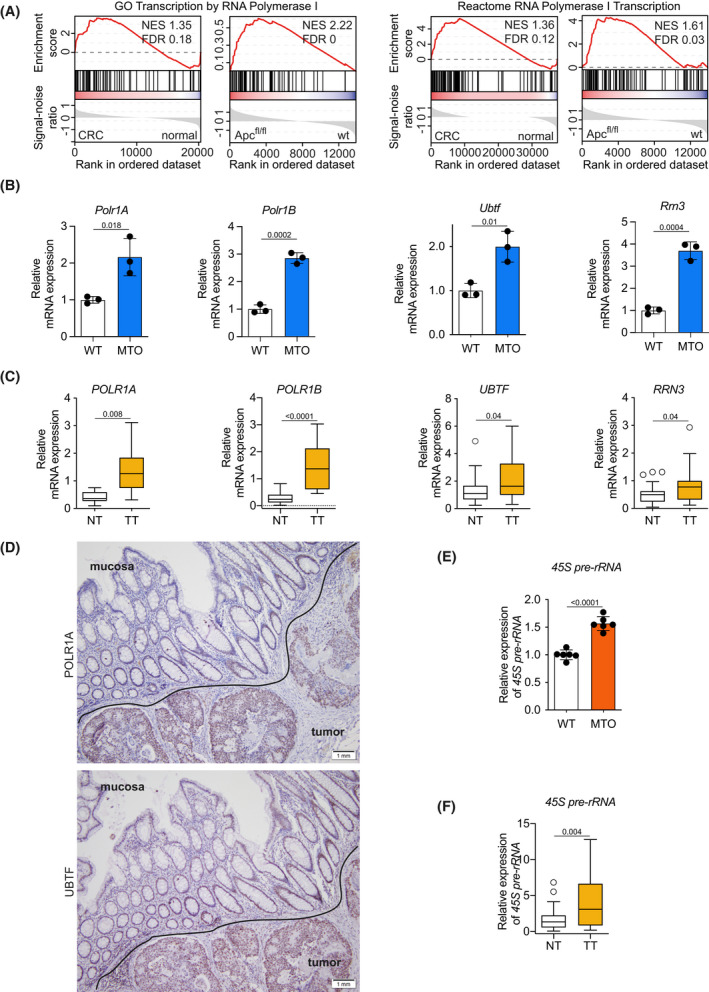
Oncogenic activation of WNT pathway upregulates RNAPOL1 machinery and its functional activity. (A) Gene Set Enrichment Analysis (GSEA) comparing human wild‐type mucosa to colorectal (CRC) samples from the ‘The Cancer Genome Atlas’ (TCGA) database (left enrichment plots) (*n* = 276) and analysis comparing intestinal mucosa from wild‐type mice to mucosa from mice with a bi‐allelic deletion of adenomatous polyposis coli (*Apc*; right enrichment plots; *n* = 3). Enrichment plots of indicated gene sets are displayed. Calculation of the normalized enrichment score (NES) is based on a weighted running sum statistic and computed as part of the GSEA methodology. A Kolmogorov–Smirnov test with 1000 permutations was used to calculate *P* values that were then corrected for multiple testing using the Benjamini–Hoechberg procedure (FDR). (B) mRNA expression of RNA polymerase 1 (RNAPOL1) subunits (*Polr1A and Polr1B*) and components of RNAPOL1 preinitiation complex (*Rrn3 and Ubtf*) in murine tumor organoids (MTOs) with *Apc* ^*def*^ *Tgfb* ^*del*^ *Kras* ^*mut*^ *Tp53* ^*del*^ relative to the expression in WT organoids analyzed via qPCR, representative of three independent experiments with similar results obtained; unpaired, two‐tailed *t*‐test; error bars indicate standard deviation (SD). (C) mRNA expression of RNAPOL1 subunits (*POLR1A and POLR1B*) and components of RNAPOL1 preinitiation complex (*RRN3 and UBTF*) in human primary CRC samples (TT) in comparison to corresponding untransformed mucosa (NT) analyzed via qPCR. Data show mean ± SD (*n* = 8 individual patient samples for POLR1A and *n* = 28 for POLR1B, RRN3 and UBFT); unpaired, two‐tailed *t*‐test. (D) Immunohistochemical staining of POLR1A and UBTF in human CRC samples and corresponding untransformed mucosa (representative images of *n* = 10 biologically independent patients). Scale bars as indicated 1 mm. (E) Expression level of 45S pre‐rRNA in MTOs compared with WT organoids determined via qPCR. Data show the mean ± SD of six technical replicates. Results are representative of three independent experiments with similar results obtained; unpaired, two‐tailed *t*‐test. (F) Expression level of 45S pre‐rRNA in human CRC tumor tissue (TT) compared with normal tissue (NT) samples determined via qPCR. Data show mean ± SD (*n* = 31 individual patient samples); unpaired, two‐tailed *t*‐test.

To identify the therapeutic vulnerabilities of CRC cells, we engineered a bi‐allelic *APC* mutated CRC (APC^def^) cell line to re‐express full‐length APC (APC^res^) in a doxycycline‐dependent manner [5]. In this cell system, restoration of APC reduces the expression of the major WNT target gene *MYC*, and the capacity for anchorage‐independent growth is impaired. RNA‐sequencing (RNA‐Seq) with subsequent Gene Set Enrichment Analysis (GSEA) comparing APC^def^ vs. APC^res^ cells showed a significant regulation of WNT‐ and MYC‐associated gene sets, as expected [5]. In addition, gene sets comprising genes encoding the RNAPOL1 machinery were expressed at significantly higher levels in APC^def^ vs. APC^res^ cells (Fig. S1A). Similarly, RNA‐Seq analysis of publicly available data comparing human wild‐type (WT) mucosa to CRC samples and analysis comparing intestinal mucosa from wild‐type mice to mucosa from mice with a bi‐allelic deletion of APC revealed a significant induction of RNAPOL1‐related gene sets in CRC and in the APC‐deleted situation (Fig. 1A and Fig. S1B) [8, 21].

To validate these findings in a well‐defined genetic setting marked by APC loss, we took advantage of murine tumor organoids (MTOs) established from murine intestinal tumors bearing deletions or mutations in the four key human CRC driver genes *Apc*, *Kras*, *Tgfbr2*, and *Trp53* [22]. We determined the expression levels of the different RNAPOL1 subunits, *Polr1a* and *Polr1b*, and essential components of its preinitiation complex, *Ubf* (upstream binding transcription factor, Ubtf), and *Rrn3*, which is necessary to direct RNAPOL1 to rDNA promoters and start transcription. MTOs showed a significant upregulation of mRNA levels of RNAPOL1 subunits and of preinitiation complex factors relative to murine intestinal WT organoids (Fig. 1B). Analyzing the expression of the RNAPOL1 subunits in murine organoids that carry only an APC deletion, we observed a comparable upregulation arguing that loss of APC and therefore aberrant WNT pathway activation alone is sufficient to drive increased RNAPOL1 machinery expression (Fig. S1C). In line with this, we also found expression of RNAPOL1 subunits and associated factors upregulated in five different CRC cell lines (DLD1, HCT116, HT29, LS174t, SW480) harboring oncogenic activation of the WNT pathway by either APC or beta‐catenin mutation in addition to additional mutations in CRC associated genes, relative to normal colon mucosa [23, 24] (Fig. S1D). Similar results were obtained when comparing human primary CRC samples (TT) to corresponding untransformed mucosa (NT; Fig. 1C). Furthermore, histopathological staining for POLR1A and UBTF in human CRC samples revealed an enhanced expression of both proteins in tumors relative to normal mucosa. As reported before, we observed a zonation of POLR1A from higher levels at the border to lower levels in the tumor center [15]. Interestingly, in addition, we also found higher POLR1A and UBTF levels at the bottom of the crypts (Fig. 1D). Consistent with these findings, human CRCs showed elevated mRNA expression of all four subunits of RNAPOL1 relative to normal mucosa (Fig. S1E).

RNPOL1 transcribes rDNA within the nucleolus, resulting in a primary transcript, which is a large precursor rRNA (47S pre‐rRNA) that is rapidly processed to a second precursor, the 45S pre‐RNA. This precursor is ultimately cleaved into three rRNA components, 18S, 5.8S, and 28S [25]. In addition to increased expression levels of RNAPOL1 subunits and preinitiation complex‐associated factors (Fig. 1B–D), we found an upregulation of 45S pre‐rRNA expression in MTOs, CRC primary tumor samples (Fig. 1E,F), and CRC cell lines (Fig. S1F). We concluded, that in CRC, due to aberrant WNT pathway activation, RNAPOL1 machinery is highly expressed, and its transcriptional activity is heightened with a consecutive overexpression of corresponding rRNAs.

**Fig. 2:**
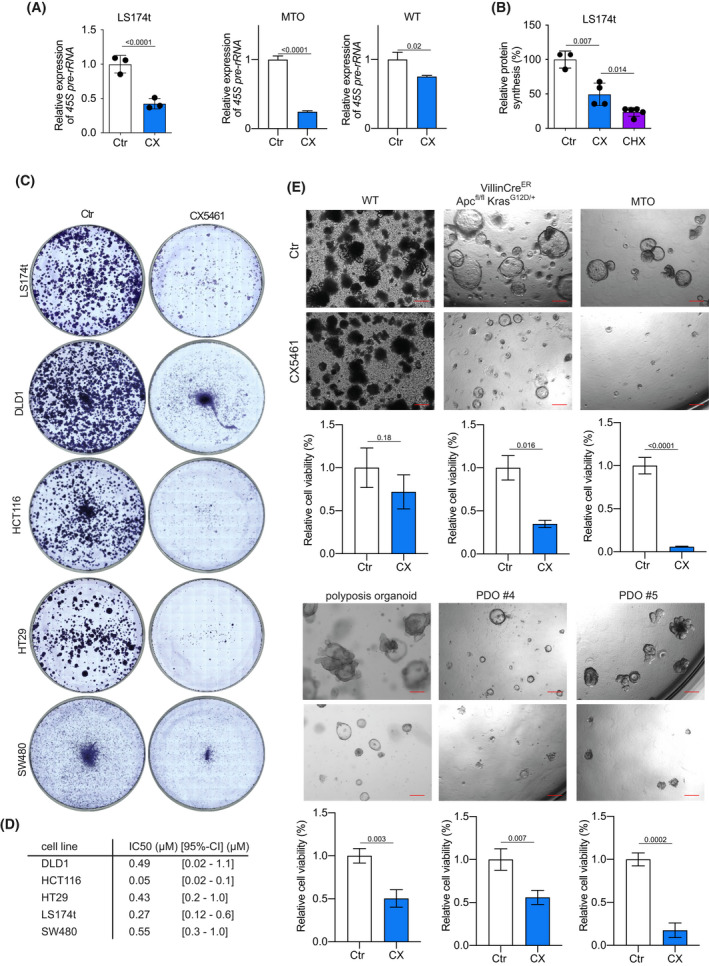
Small molecule CX5461 inhibits transcriptional activity of RNAPOL1 and impairs viability of CRC cells. (A) Expression level of 45S pre‐rRNA in LS174t cells (left plot) after 24 h of CX5461 treatment (CX, 500 nm) or treatment with a comparable volume of control vehicle (Ctr) analyzed via qPCR. Results are representative of three independent experiments, with similar results obtained; unpaired, two‐tailed *t*‐test; error bars indicate SD. Expression level of 45S pre‐rRNA in MTO (middle plot) or murine WT organoids (right plot) after 24 h of CX5461 (CX, 500 nm) treatment or treatment with a comparable volume of control vehicle (Ctr) analyzed via qPCR. Data show the mean of at least four biological replicates of each organoid line; unpaired, two‐tailed *t*‐test, error bars indicate SD. (B) Quantitative analysis of the immunofluorescence of O‐propargyl‐puromycin (OPP) incorporation to measure protein synthesis rate in LS174t cells treated with control vehicle (Ctr) and CX5461 (CX, 500 nm) for 3 days. Results are representative of three independent experiments with similar results obtained; unpaired, two‐tailed *t*‐test, error bars indicate SD. (C) Crystal violet staining of LS174t, HCT116, DLD1, HT29, and SW480 cells after 5 days of treatment with CX5461 (500 nm) or a comparable volume of control vehicle. Images are representative of three independent experiments with similar results obtained. (D) Table showing IC50 of indicated cell lines determined via WST‐8 assay. (E) Growth of organoids after treatment with CX5461. WT organoids, VillinCre^ER^ Apc^fl/fl^ Kras^G12D/+^ organoids, MTOs, polyposis organoids, and two lines of patient‐derived tumor organoids (PDOs, #4 and #5) were grown for at least 12 h, then treated with CX5461 (CX, 250 or 500 nm, respectively) or control vehicle for at least 72 h. Upper panel: Representative pictures of one organoid line of each genotype. Scale bars, 200 μm. Lower panel: Viability of organoids treated as described. Assessed using CellTiter‐Blue assay. Data show the mean of four biological replicates of WT, VillinCre^ER^ Apc^fl/fl^ Kras^G12D/+^, and MTO organoids and five biological replicates for polyposis organoid, PDO #4 and #5, error bars indicate SD.

The increased transcriptional activity of RNAPOL1 machinery driven by activated WNT signaling implicates that inhibition of RNAPOL1 may offer a therapeutic window in CRC. Therefore, we studied the consequences of inhibition of RNAPOL1 transcription by taking advantage of the well‐characterized RNAPOL1 inhibitor CX5461 [26]. This small molecule is supposed, besides other modes of action, to inhibit RNAPOL1 transcription by perturbing the recruitment of SL1 to chromatin; SL1 is a multi‐component factor of the preinitiation complex that is necessary to direct RNAPOL1 to rDNA [26]. Treatment of LS174t cells with CX5461 for 24 h significantly reduced rDNA transcription as measured by 45S pre‐RNA expression (Fig. 2A). These findings were corroborated in MTOs in which levels of 45S pre‐rRNA were reduced by approximately 80% upon CX5461 treatment. In contrast, WT organoids show only a slight decrease in 45S pre‐rRNA levels after CX5461 treatment (Fig. 2A). The activity of RNAPOL1, quantified by the abundance of the 45S pre‐rRNA, is an important indicator of global protein synthesis rates [27]. Correspondingly, O‐propargyl‐puromycin incorporation into newly synthesized proteins was significantly reduced after treatment with CX5461 (Fig. 2B).

To show that CX5461 is selective for RNAPOL1, phosphorylation of serine 2 of RPB1, the largest subunit of the RNAPOLII complex, was determined by immunoblotting. The C‐terminal domain of RNAPOLII contains several repeats of a heptameric structure (YSPTSPS). While phosphorylation of serine 5 within these repeats is associated with RNAPOLII transcription initiation, serine 2 is phosphorylated during promoter‐proximal pause release and RNAPOLII elongation [28]. The treatment with THZ1, a selective CDK7 inhibitor, clearly reduced phosphorylation of serine 2, while treatment with CX5461 did not (Fig. S2A). In addition, we evaluated the effect of CX5461 on RNAPOLIII, which transcribes a range of short noncoding RNAs including 5S rRNA and 7SL RNA. Incubation with CX5461 for 24 h did not change 5S rRNA and 7SL RNA levels (Fig. S2B).

Next, we tested the impact of CX5461 on the viability of several CRC cell lines harboring different mutations, and murine and patient‐derived organoids. Treatment with CX5461 dose‐dependently decreased cell viability in all cell lines tested with IC50 values in the nanomolar range (Fig. 2C,D). Whereas CX5461 treatment had minor effects on the viability of organoids established from WT mucosa, it dramatically reduced the growth of murine VillinCre^ER^Apc^fl/fl^ Kras^G12D/+^ organoids and MTOs (Fig. 2E). In line with this, CX5461 treatment of organoids established from a patient harboring a germline APC mutation, and two patient‐derived tumor organoids (PDO) showed significant growth inhibition (Fig. 2E). Together, these results indicate that RNAPOL1 inhibition by CX5461 may open a therapeutic window in CRC.

**Fig. 3:**
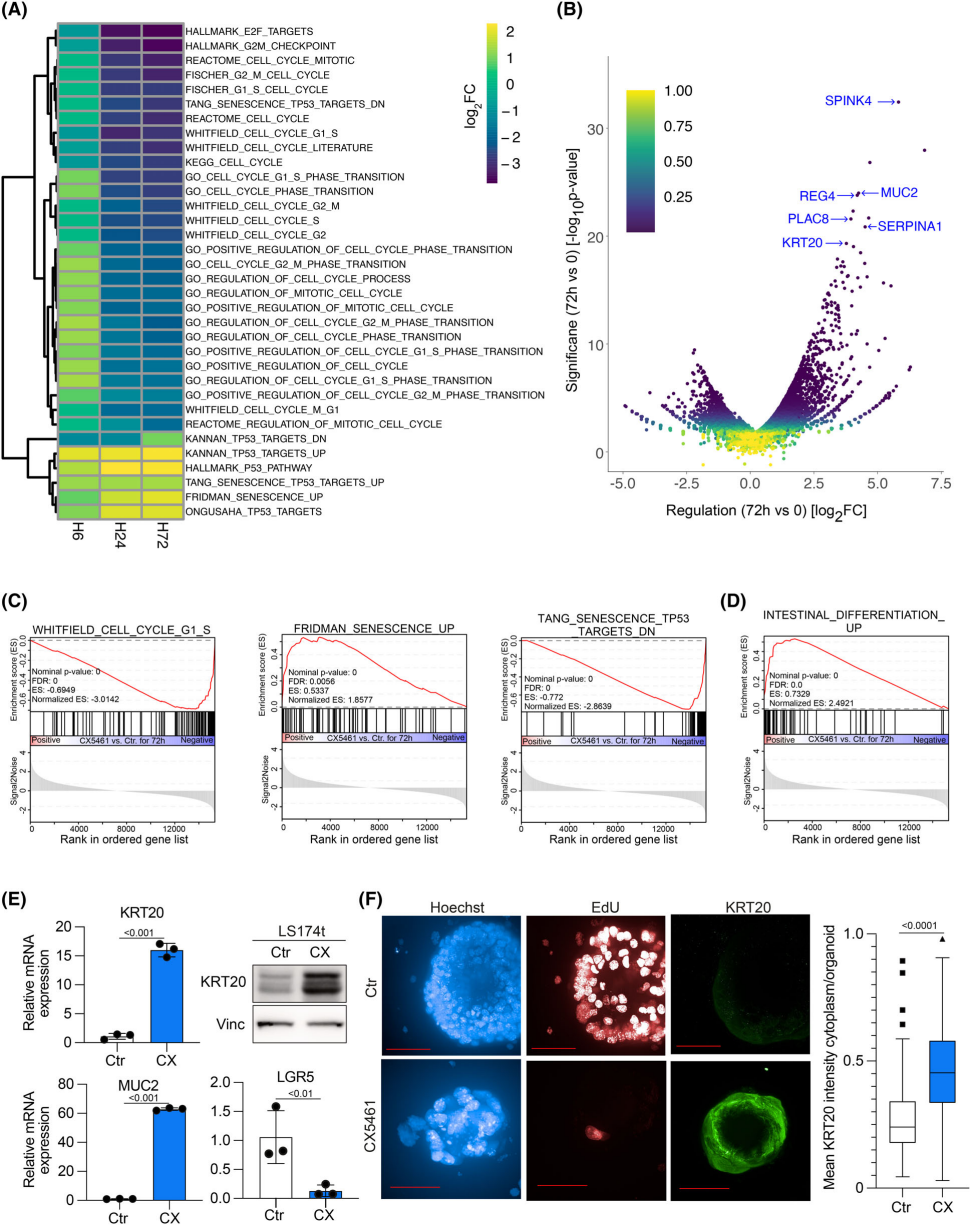
CX5461 induces differentiation and features of senescence in CRC cells. (A) RNA‐Seq of LS174t cells treated with CX5461 or control vehicle for 6, 24, and 72 h. Heatmap depicting regulated gene sets (*n* = 3 biological replicates). (B) Volcano plot comparing RNA‐Seq results of 72 h CX5461 treatment to Ctr samples by plotting log_2_FC on the *x*‐axis and −log10*P*‐value (significance of change in expression) on *y*‐axis. (C) GSEA enrichment plots of annotated gene sets for cell cycle and senescence in CX5461 treated cells (72 h) in comparison to control samples. (D) GSEA enrichment plots of a gene set comprising differentiation‐associated genes of intestinal mucosa/CRC in CX5461 treated cells (72 h) in comparison to control samples. (E) mRNA expression of *KRT20*, *MUC2*, and *LGR5* (left upper and lower panels) in CX5461 (500 nm for 72 h) treated LS174t cells relative to cells treated with a control vehicle; immunoblot of KRT20 (right upper panel) in LS174t cells treated with CX5461 (500 nm for 72 h) or control vehicle. Data show mean ± SD. Results are representative of three independent experiments, with similar results obtained; unpaired, two‐tailed *t*‐test. (F) Left panel: Immunofluorescence staining for Hoechst, EdU, and KRT20 in MTOs treated with CX5461 (500 nm for 96 h); scale bar 50 μm; Right panel: Quantification of KRT20 staining in MTOs comparing CX5461 treatment condition to control the situation (*n* = 50 individual organoids); unpaired, two‐tailed *t*‐test.

To evaluate the impact of CX5461 on gene expression, we performed RNA‐Seq from LS174t cells incubated with CX5461 for 6, 24, or 72 h. GSEA showed an enrichment of genes associated with cell cycle arrest, a differentiated, intestinal cell phenotype, and features of senescence upon CX5461 treatment (Fig. 3A). Conversely, gene sets of cell cycle progression were suppressed. These shifts in gene expression became more prominent over time; in particular, the increase in expression of differentiation‐associated genes mainly emerged after 72 h of treatment (Fig. 3A–C). As differentiation‐associated genes were one of the most significantly upregulated genes upon CX5461 treatment (Fig. 3B), we compiled two new gene sets of published markers for the characterization of intestinal differentiation state or stemness within intestinal mucosa, respectively. In an unbiased manner, we identified 93 potential genes, which characterize the specific cell types present within the intestinal mucosa. Expression of the differentiation gene set is highly enriched in WT mucosa vs. mucosa after APC depletion (Apc^fl/fl^; Fig. S3A,B). Vice versa, the stemness‐defining gene set is upregulated after the loss of APC. Applying these gene sets on LS174t cells uncovered that treatment with the RNAPOL1 inhibitor CX5461 induced a differentiated cellular phenotype (Fig. 3D). From these data, we concluded that treatment with CX5461 may induce differentiation in CRC cells.

Two well‐described markers of differentiation in CRC—KRT20 and MUC2—scored in the top 1% of upregulated genes upon CX5461 treatment (Fig. 3B). We validated these findings from RNA‐Seq followed by GSEA on mRNA and protein levels. In this context, treatment of LS174t cells with CX5461 for 72 h led to a strong upregulation of KRT20 and MUC2, while LGR5 was significantly downregulated (Fig. 3E). Furthermore, we analyzed the effect of CX5461 treatment on MTOs. While organoids treated with a control vehicle showed normal EdU incorporation as a marker of undisturbed cell cycle progression and only slight staining for KRT20, strikingly, CX5461 treatment for 72 h completely abolished EdU incorporation and induced strong staining for KRT20 (Fig. 3F). In addition, four CRC cell lines showed comparable upregulation of KRT20 and MUC2 after 72 h (Fig. S3C).

**Fig. 4:**
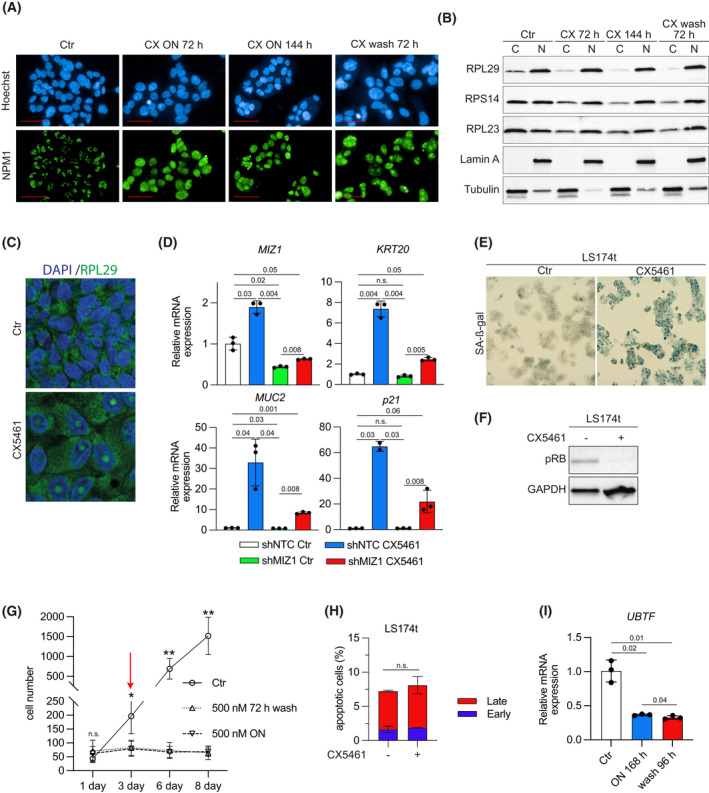
CX5461 induces terminal differentiation in a MIZ1‐dependent manner. (A) Immunofluorescence of LS174t treated with CX5461 (CX, 500 nm) or control vehicle for the indicated time (CX ON: CX5461 treatment for the indicated time in hours; CX wash: CX5461 treatment for 72 h followed by incubation for further 72 h after inhibitor washout), scale bar 50 μm. Images are representative of three independent experiments with similar results obtained. (B) Immunoblot of LS174t cells treated with CX5461 or control vehicle for indicated proteins after nuclear/cytoplasmic fractionation. The blot is representative of three independent experiments with similar results obtained. (C) Immunofluorescence of LS174t stained for ribosomal protein L29 (RPL29) and 4′,6‐diamidino‐2‐phenylindole (DAPI). Treatment for 7 days with CX5461 (500 nm) or a control vehicle. Images (100× magnification) are representative of two independent experiments with similar results obtained. (D) mRNA expression of *MIZ1*, *KRT20*, *MUC2*, and *CDKN1A* (p21) of shCTR transduced or MIZ1‐depleted LS174t cells after treatment with CX5461 (500 nm for 72 h) or control vehicle. Data show mean ± SD. Results are representative of three independent experiments with similar results obtained; unpaired, two‐tailed *t*‐test. (E) Staining of LS174t cells for senescence‐associated β‐galactosidase activity after 7 days of treatment with CX5461 (500 nm) or control vehicle. Images (4× magnification) are representative of three independent experiments with similar results obtained. (F) Immunoblot of LS174t cells treated with CX5461 (500 nm for 7 days) or a control vehicle for indicated proteins. The blot is representative of two independent experiments with similar results obtained. (G) Growth curve of LS174t treated with CX5461 (500 nm) or a control vehicle. ‘ON’ marks the curve of cells treated with CX5461 all the time. Seventy‐two hours wash cells are initially treated with CX5461 for 72 h followed by an inhibitor washout and further incubation for indicated time without inhibitor. Timepoint 3 days (red arrow) marks the timepoint of treatment removal and change to inhibitor‐free medium when indicated (wash). Assessment of cell number was done every 24 h (*n* = 6 independent biological replicates for Ctr, *n* = 8 independent biological replicates for CX5461‐treated cells; error bars indicate SD (* = *P* < 0.01; ** = *P* < 0.001). (H) Annexin V/PI FACS analysis of LS174t cells treated with CX5461 (500 nm) or a control vehicle for 24 h. Early apoptotic cells were characterized by single annexin V staining; late apoptotic cells were characterized by staining for annexin V and PI. Data show mean ± SD. Results are representative of three independent experiments with similar results obtained; unpaired, two‐tailed *t*‐test. (I) mRNA expression of *UBTF* in LS174t treated with CX5461 (CX, 500 nm) for 168 h or treated for 72 h before further incubation without inhibitor for another 96 h relative to the control vehicle. Data show mean ± SD. Results are representative of two independent experiments with similar results obtained; unpaired, two‐tailed *t*‐test.

Disturbances in ribosomal biogenesis either by lack of rRNA components or individual ribosomal proteins can lead to nucleolar disruption [29]. Nucleophosmin (NPM1) is a highly abundant and multifunctional protein mainly located in the granular component of the nucleolus that can shuttle to the nucleus and cytoplasm triggered by nucleolar stress [30, 31]. Immunofluorescence staining of CX5461‐treated LS174t cells showed a loss of the physiological granular nucleoli structure (seen in control cells), an increase of NPM1 staining in the entire nucleoplasm, and an aggregation of one or two NPM1‐stained larger structures within the nucleus, indicating disruption of nucleolar integrity upon CX5461 treatment (Fig. 4A). In line with the disruption of the nucleolus, the nuclear to cytoplasmic ratio of RPL23, RPL29, and RPS14 was significantly altered under CX5461 treatment (Fig. 4B), indicating that a reduction in rRNA synthesis leads to an alteration in the localization of these proteins. Correspondingly, immunofluorescent staining of the ribosomal protein RPL29 revealed its accumulation within large singular structures in the nucleus (Fig. 4C). Remarkably, NPM1 re‐location and nuclear aggregation of ribosomal proteins were only partially reverted after inhibitor washout for several days (Fig. 4A,B).

An imbalance of ribosomal biogenesis and the resulting re‐localization of NPM1 to the nucleoplasm induces MIZ1, a transcription factor with activating and suppressive functions depending on its binding partner [15, 32, 33, 34, 35]. MIZ1 has established roles in inducing p21CIP1 upregulation, cell cycle arrest, and cell differentiation [32, 36]. As treatment with CX5461 led to a strong induction of KRT20 and MUC2, we checked for another well‐characterized MIZ1‐associated cell cycle inhibitor (p21CIP1). In line with previous data, CX5461 led to a strong induction of p21 on mRNA and protein levels (Fig. S4A). Genetic depletion of MIZ1 using shRNAs targeting MIZ1 reduced its expression by approximately 50%. Strikingly, this knockdown significantly reduced the induction of KRT20, MUC2, and p21CIP1 upon CX5461 treatment (Fig. 4D). From these data, we conclude that CX5461 treatment‐associated induction of differentiation is at least partially mediated via MIZ1 in CRC.

In addition to cell differentiation and in line with the GSEA data showing induction of gene sets related to senescence, treatment with CX5461 for 7 days led to a strong increase in SA‐β‐gal staining (Fig. 4E). Furthermore, we observed additional markers of cell cycle arrest and senescence such as dephosphorylation of the mitotic marker p‐histone H3 (Ser10), loss of the E2F target MCM6 [37], and accumulation of cyclin D1 [38] (Fig. S4B) [39]. These markers were also found in MTOs (Fig. S4C). Four additional tested CRC cell lines also showed an increase in SA‐β‐gal staining after 7 days of treatment (Fig. S4D). According to previous reports that accumulation of free ribosomal proteins can induce senescence via the RB pathway, we observed a reduction in RB phosphorylation (Fig. 4F), an induction of RB gene sets within the GSEA after CX5461 treatment, and a reduced expression of MCM6, a downstream target of E2F signaling, which is negatively regulated by RB (Fig. S5A,B), indicating an activation of this signaling pathway with subsequent negative control of the cell cycle [32].

Phenotypically, treatment with 500 nm CX5461 reduced cell growth and suppressed colony formation leading to terminal differentiation and features of senescence that persisted even after washout. As proof of terminal differentiation and inability to resume normal cell cycle, we incubated LS174t cells with 500 nm CX5461 for 72 h followed by a washout of the inhibitor and further incubation for several days. Strikingly, while cells treated with vehicle showed continuous cell growth, cells initially treated with CX5461 for 72 h did not resume proliferation even 5 days after washout of the inhibitor (Fig. 4G). As previously described, while we observed a cell cycle arrest, we did not see an induction of apoptosis upon CX5461 treatment in CRC cells (Fig. 4G,H). In line with the previously suggested model, that the ability of protein translation prevents differentiation, neither rDNA transcription nor protein translation was significantly restored after inhibitor washout (Fig. S5C,E). To further evaluate the observed continuous suppression of rDNA transcription we checked for the expression of RNAPOL1 components within the RNA‐Seq analysis after CX5461 treatment. While the expression of several components did not change, including POLR1A, approximal 75% are downregulated including UBTF (Fig. S5F). Strikingly, RTQ‐PCR showed a constant reduction in UBTF mRNA expression levels even after CX5461 washout for several days (Fig. 4I). In conclusion, these data argue that interfering with rDNA transcription via CX5461 leads to a differentiated cellular phenotype and induces features of senescence in a cellular setting of altered WNT pathway activity.

**Fig. 5:**
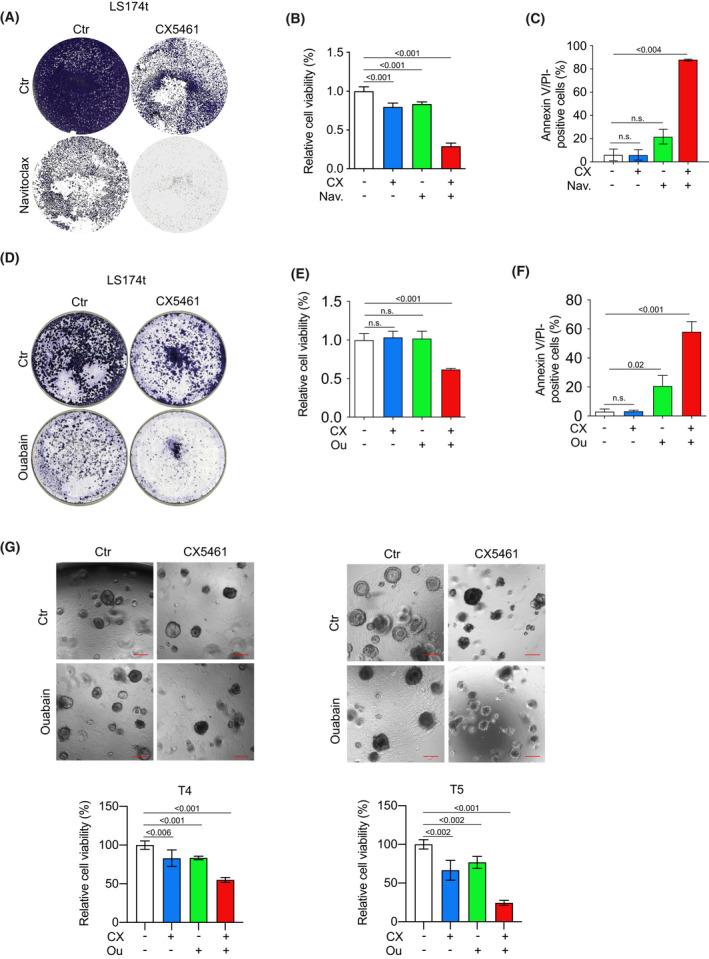
Sequential treatment of CRC cells with RNAPOLI inhibitor followed by a senolytic drug shows additive effects on cell viability. (A) Crystal violet staining of LS174t cells treated with control vehicle, CX5461 (500 nm) or Navitoclax (500 nm) as a single treatment or CX5461 and Navitoclax as a sequential treatment for 7 days. Images are representative of three independent experiments with similar results obtained. (B) Quantification of cell viability of LS174t cells treated with a control vehicle, CX5461 alone, Navitoclax alone or sequentially with CX5461 followed by a Navitoclax application using WST‐8 assay. Data show the mean of at least three technical replicates. Data are representative of three biologically independent experiments with similar results obtained; unpaired, two‐tailed *t*‐test; error bars indicate SD. (C) Annexin V/PI FACS analysis of LS174t cells treated with a control vehicle, CX5461 alone (CX, 500 nm), Navitoclax alone (N, 500 nm) or sequentially with CX5461 followed by an additional application of Navitoclax for in total 7 days. Data show mean ± SD. Results are representative of three independent experiments with similar results obtained; unpaired, two‐tailed *t*‐test. (D) Crystal violet staining of LS174t cells treated with control vehicle, CX5461 (500 nm) or ouabain (500 nm) as a single treatment or CX5461 and ouabain as a sequential treatment for 7 days. Images are representative of three independent experiments with similar results obtained. (E) Quantification of cell viability of LS174t cells treated with a control vehicle, CX5461 (CX, 500 nm) alone, ouabain (Ou, 500 nm) alone or sequentially with CX5461 followed by additional application ouabain for 3 days using WST‐8 Assay. Data show the mean of at least three technical replicates. Data are representative of three biologically independent experiments with similar results obtained; unpaired, two‐tailed *t*‐test; error bars indicate SD. (F) Annexin V/PI FACS analysis of LS174t cells treated with a control vehicle, CX5461 (CX, 500 nm) alone, ouabain (Ou, 500 nm) alone or sequentially with CX5461 followed by an additional application of ouabain for in total 7 days. Data show the mean ± SD of three independent experiments; unpaired, two‐tailed *t*‐test. (G) Growth of PDO #4 and #5 after treatment with control vehicle, CX5461 (CX, 100 nm) single treatment, ouabain (Ou, 250 nm) single treatment or sequential treatment of CX5461 and ouabain. Treatment was started 2 h after seeding. Additional ouabain was added 3 days after the initial CX5461 treatment. Upper panel: Representative pictures. Scale bars, 200 μm. Lower panel: Viability of organoids treated as described assessed using CellTiter‐Blue assay. Data show the mean of four technical replicates for PDO #4 and six technical replicates for #5. Data are representative of at least three biologically independent organoid lines per genotype and experiments with similar results obtained; unpaired, two‐tailed *t*‐test, error bars indicate SD.

In our study, CX5461 induces both a differentiated cellular phenotype and features of senescence, but viable tumor cells remained after treatment. Therefore, we asked whether this induced phenotype could be exploited as a vulnerability by combinatory usage of agents that specifically kill cells with features of senescence and that have proven beneficial effects in several age‐related diseases [40]. Currently, Bcl‐2 family inhibitors like ABT‐263 (Navitoclax) are the most widely used drugs in this class [41]. To test our hypothesis, we treated LS714t cells with either CX5461 or navitoclax as a single treatment, or with both drugs combined in a sequential manner (Fig. 5A–C). Whereas single treatment with each drug had only marginal effects on cell viability, the sequential combination of both led to a significant reduction in cell viability (Fig. 5B,C). Consistent with previous data, treatment with CX5461 did not increase the percentage of cells positive for both annexin V and propidium iodide (PI), whereas navitoclax alone had a modest effect (Fig. 5B). Strikingly, consecutive treatment with both drugs led to a strong induction of apoptosis with over 80% annexin V/PI‐positive cells (Fig. 5C). We further validated the potential of combinatory treatment in four other cell lines. Comparable results were found validating that the combinational treatment has dramatic synergistic effects (Fig. S6A). This data argues that the CX5461‐induced growth arrest can have therapeutic potential in combination with drugs using this vulnerability in treating CRC. As the use of navitoclax is limited due to its adverse side effects such as thrombopenia, we took advantage of the recent observation that cardiac glycosides can induce senescence with few side effects [42, 43]. Ouabain is one of the best characterized cardiac glycosides that triggers cell death in oncogene‐induced and in therapy‐induced senescent cells [42]. Whereas treatment with ouabain alone had only marginal effects, it induced strong antiproliferative effects in cells pretreated with CX5461 (Fig. 5D,E and Fig. S5B). In line with these results, we also observed significant additive effects of CX5461 and Ouabain in human PDOs (T4 and T5; Fig. 5G).

**Fig. 6:**
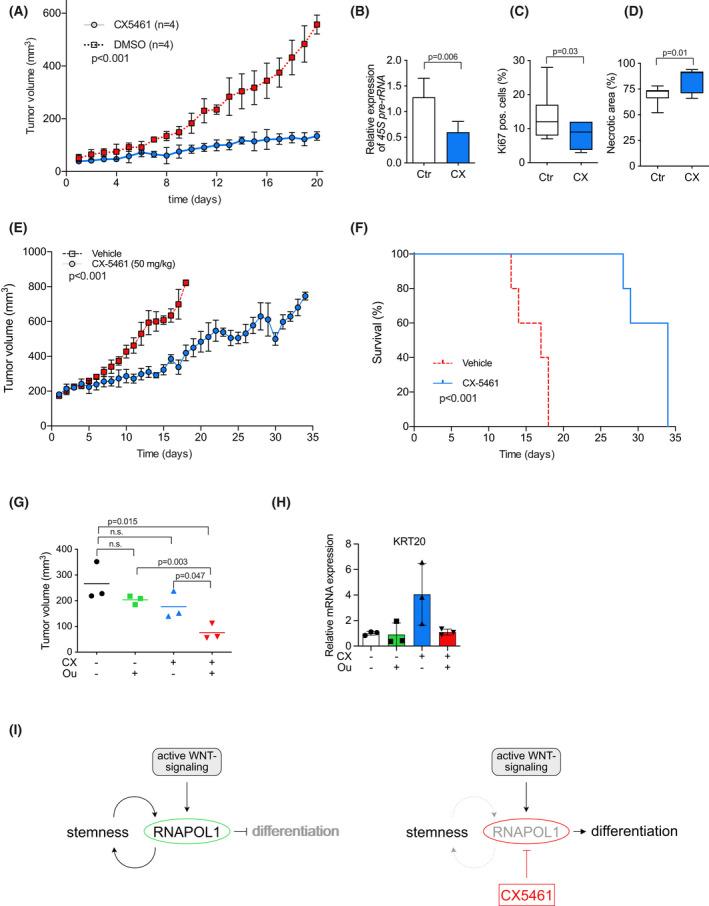
CX5461 inhibits tumor growth *in vivo* and additional treatment with ouabain has additional effects due to the clearing of senescent tumor cells. (A) Quantification of daily‐assessed tumor growth in a xenograft tumor model. When tumor size reached 50–60 mm^3^ after subcutaneous injection of tumor cells mice were randomized in a CX5461 (50 mg·kg^−1^ orally) or control vehicle treatment group. Two‐way ANOVA; data show mean ± SD of *n* = 4 mice per group. (B) Expression level of 45S pre‐rRNA in tumor tissue from mice described in (A) comparing CX5461 (CX, 50 mg·kg^−1^ orally) to control vehicle (Ctr) group analyzed by qPCR. Data show mean ± SD; unpaired, two‐tailed *t*‐test. (C) Quantitative analysis of the immunohistochemical staining for Ki67 in tumor xenografts by comparing CX5461 (CX, 50 mg·kg^−1^ orally) and control vehicle (Ctr) treated mice described in (A). Data show mean ± SD, unpaired, two‐tailed *t*‐test. (D) Quantitative analysis of the necrotic area in tumor xenografts by comparing CX5461 (CX) and control vehicle (Ctr) treated mice described in (A). Data show mean ± SD; unpaired, two‐tailed *t*‐test. (E) Quantification of daily‐assessed tumor growth in a xenograft tumor model. When tumor size reached 200 mm^3^ after subcutaneous injection of tumor cells mice were randomized in a CX5461 (CX, 50 mg·kg^−1^ orally) or control vehicle (Ctr) treatment group. Data show mean ± SD of *n* = 5 mice per group. Two‐way ANOVA. (F) Kaplan–Meier‐curve depicting survival of mice described in (E); Kaplan–Meier survival analysis. (G) Quantification of final xenograft tumor volume to show an increase in antitumor effect by the combination of CX5461 (CX) and ouabain (Ou). When tumor growth reached 100 mm^3^ mice were randomly assigned to a control group (*n* = 3), to an ouabain (1 mg·kg^−1^ i.p.) treatment group (*n* = 3) or to low‐dose CX5461 (30 mg·kg^−1^ orally) treatment group (*n* = 6). After 7 days, half of the CX5461‐treated mice received an additional 3 days of ouabain (*n* = 3); unpaired, two‐tailed *t*‐test; horizontal lines indicate mean. (H) mRNA expression level of *KRT20* in tumor tissue from mice described in (G). analyzed by qPCR (*n* = 3 mice); unpaired, two‐tailed *t*‐test, error bars indicate SD. Model explaining our findings. Active WNT signaling induces RNAPOL1 preventing differentiation and keeping stemness. Treatment with an RNAPOL1 inhibitor like CX5461 disrupts this feed‐forward loop and induces differentiation.

We next examined whether inhibition of RNAPOL1 transcription by CX5461 is effective for the treatment of CRC tumors *in vivo*. To this end, we used a well‐characterized xenograft tumor model [20]. Two million cells were injected subcutaneously into immune‐deficient nude mice. Tumor volume was determined on daily basis. When a tumor volume of 50 mm^3^ was reached, mice were randomly assigned to a single oral dose of CX5461 (50 mg·kg^−1^) per day or vehicle control. Whereas tumors in control mice showed an exponential growth curve over time, reaching the cut‐off volume of around 600 mm^3^ 20 days after randomization, tumors of CX5461‐treated mice showed strongly retarded tumor growth (Fig. 6A). To exclude cytotoxic effects of CX5461 treatment, we additionally measured several physiological parameters. Body weight, liver enzyme levels, and leukocytes and thrombocytes did not differ between CX5461‐treated and control groups, whereas red blood cell count and hemoglobin (Hb) showed a slight decrease after treatment with CX5461 (Fig. S6A,B). In tumors from mice treated with CX5461, we observed reduced expression of 45S pre‐rRNA, arguing that CX5461 is on target in the desired tissue area and successfully hits the tumor cells (Fig. 6B). In addition, tumors from CX5461‐treated mice had significantly reduced Ki67‐positive cells and significantly higher necrosis area (Fig. 6C,D).

As CX5461 treatment blocked the growth of early tumors (Fig. 6A), we were also interested in whether CX5461 also has a therapeutic impact on advanced tumors. To evaluate this, we allowed tumors to grow to a volume of 200 mm^3^ and then randomized mice into control and CX5461 treatment groups. Whereas control tumors showed an exponential growth curve reaching the cut‐off value after 15 days, CX5461 treatment significantly limited tumor growth and increased the median survival time from 18 to 34 days (Fig. 6E,F).

Finally, we tested if treatment of CX5461 and subsequent treatment with ouabain also has a synergistic effect *in vivo*. When tumor growth reached 100 mm^3^ mice were randomly assigned to a control group (*n* = 3), to an ouabain treatment group (*n* = 3) or to a low‐dose CX5461 treatment group (*n* = 6). After 7 days, half of the CX5461‐treated mice received an additional 3 days of ouabain (*n* = 3). Strikingly, while treatment with low‐dose CX5461 or ouabain had only slight effects, ouabain application to CX5461‐pretreated tumors for 3 days significantly reduced tumor volume (*P* = 0.047; Fig. 6G). While control vehicle and ouabain‐treated tumors show similar levels of KRT20, this significantly increased with CX5461 treatment. Intriguingly, ouabain completely abolished the CX5461‐induced upregulation of KRT20, arguing that ouabain eliminates the differentiated/senescent cells induced by CX5461 treatment *in vivo* (Fig. 6H).

## 4 Discussion

Activation of WNT signaling increases global protein synthesis and induces the protein transcription machinery [6]. Using murine organoids with a defined genotype and patient‐derived organoids from CRC, we uncovered a significant upregulation of rDNA transcription to balance ribosome formation. Furthermore, we show that disruption of this balance leads to cellular differentiation with features of senescence in a MIZ1‐dependent manner, that can be targeted by small molecules to induce apoptosis selectively in CRC cells.

Protein translation is tightly controlled to maintain cellular homeostasis. A hallmark of tumor cells is an enhanced biosynthetic machinery, including upregulated rDNA transcription, ribosomal biogenesis, and protein synthesis rates. While protein translation in normal tissue withstands severe disturbances, it is strictly balanced in tumor tissue and even slight changes can lead to reduced cell growth or death. In line with this, several studies have shown that mice that are haplo‐insufficient/hypomorphic for translation initiation factors or ribosomal proteins or in which these have been inhibited by small molecules show normal physiology but dramatically reduced tumor growth [2, 3, 5, 7].

Recently, it has been shown that there is a heterogenous capacity for protein translation in CRC leading to zonation of proliferating, and protein‐synthesizing and silent cells. This zonation is marked by the expression of RNAPOL1/POLR1A and functionally defines cell stemness independent of LGR5. Genetic depletion of POLR1A or treatment with a POLR1A degrader induces loss of stemness in CRCs and leads to an inability to form tumors *in vivo* [15].

We now translate these basic findings into a positive feed‐forward loop with even short interruptions leading to a long‐lasting therapeutic effect.

Several reports have suggested that rRNA synthesis is enhanced in malignant tissue including CRC [16, 44]. Strikingly, we show now that components of rDNA transcription machinery and 45S pre‐rRNA are elevated in CRC due to ubiquitous activation of the WNT signaling pathway. These high RNAPOL1 machinery levels prevent differentiation and keep stemness. A short‐term interruption of RNAPOL1 activity by the usage of CX5461 is sufficient to induce differentiation in cells with persisting aberrant WNT pathway activation. Once this differentiation is induced, cells seem to be unable to re‐enter the normal cell cycle even after inhibitor washout, which at least partially restores nucleolus formation.

In line with the fact that RNAPOL1 machinery defines stemness, we observed high UBTF and POLR1A expression within the bottom of crypts in normal mucosa, the stem cell compartment and only a low expression within the upper part of the crypts.

Differentiation and senescence are suggested to be possible goals for cancer therapeutics as chemotherapy or radiotherapy. Our data now show that the small molecule CX5461 has terminal differentiation as a therapeutic endpoint in CRC. In addition, we found features of senescence as a cellular phenotype after chronic treatment with CX5461 comparable to the findings of Mars et al. [45]. So far, genetic loss of POLR1A has not been proven to be linked to senescence.

Clearance of tumor cells showing features of senescence has been proposed to improve therapeutic outcomes [46]. We also confirm here that ouabain, a cardiac glycoside (CG), acts in this way as previously suggested in age‐related senescence [42, 43]. Besides ouabain, there are several structurally related CGs such as digitoxin and digoxin, which are in daily clinical use. Interestingly, it has been shown that digoxin can inhibit tumor growth in mice [47]. In line with this, retrospective population‐based studies also suggest a positive effect of CGs in solid cancer patients [48].

The findings reported here have broader implications. It has been shown that the oncoprotein MYC is the main downstream target of activated WNT signaling and is essential to maintain CRC tumor growth [10, 36]. Virtually all tumors besides CRC display deregulated MYC expression and activity, arguing that approaches to identify critical pathways in MYC‐driven cancers have enormous therapeutic potential. Taking this into consideration, it can be speculated that targeting rDNA transcription to induce a vulnerable cellular phenotype with subsequent application of senolytics can have widespread applicability.

Our results are in line with the use of CX5461 in other cancer entities. It has been shown that CX5461 induces cell growth arrest and senescence in a p53‐dependent manner. Although we observe the same phenotype, it seems to be p53‐independent as we found it in both p53 wild‐type and p53‐deleted/−mutated background [16, 26]. In addition, it has recently been shown that an accelerated tumor development in Apc^min^ mice lacking the zinc‐finger protein 545, a transcriptional repressor for rRNA synthesis, can be reverted by CX5461 [49]. Besides the mechanism proposed by us, that CX5461 reduces rRNA synthesis, there are additional reports that suggest how CX5461 functions, e.g., by poisoning topoisomerase II or by stabilizing G‐quadruplex DNA structures and, therefore, causing DNA damage [50, 51, 52].

## 5 Conclusions

In conclusion, we showed that activated WNT signaling upregulates rDNA transcription machinery, which renders CRC highly vulnerable to RNAPOL1 inhibition. RNAPOL1 inhibition by the small molecule CX5461 in CRC leads to a cellular phenotype marked by differentiation and features of senescence, which can be further targeted by senolytic drugs to completely clear the tumor (Fig. 6I).

## Conflict of interest

The authors declare no conflict of interest.

## Author contributions

CO, CK, SS, AR, C‐TG, EW, ME, and AW conceived and designed the project. CO, CK, SS, KU, AB, SD, MTR, FR, CPA CS‐V, MED, and AW acquired the data. CO, CK, SS, AB, AR, FR, CPA, MED, C‐TG, EW, ME, and AW analyzed and interpreted the data. CO, CK, SS, C‐TG, EW, ME, and AW wrote the paper.

### Peer Review

The peer review history for this article is available at https://publons.com/publon/10.1002/1878‐0261.13265.

## Supporting information


**Fig. S1.** Oncogenic activation of WNT pathway upregulates RNAPOL1 machinery and its functional activity. (A) RNA‐Seq followed by GSEA of gene expression changes in APC^def^ and APC^res^ cells (48 h ethanol and doxycycline treatment, respectively). Enrichment plots of indicated gene sets are displayed. Calculation of the normalized enrichment score (NES) is based on a weighted running sum statistic and computed as part of the GSEA methodology. A Kolmogorov–Smirnov test with 1000 permutations was used to calculate *P* values that were then corrected for multiple testing using the Benjamini–Hochberg procedure (FDR). (B) RNA‐Seq analysis comparing intestinal mucosa from WT mice to mucosa from mice with a bi‐allelic deletion of APC or APC^def^ and APC^res^ cells (48 h ethanol and doxycycline treatment, respectively), and analysis from the TCGA database comparing human WT mucosa with CRC samples. (C) mRNA expression of RNAPOL1 subunits (*Polr1a* and *Polr1b*) and components of RNAPOL1 preinitiation complex (*Rrn3* and *Ubtf*) in Apc^fl/fl^ murine organoids relative to the expression level in WT organoids analyzed via qPCR. Data show mean ± SD of technical replicates. The results are representative of 3 independent experiments with similar results obtained; unpaired, two‐tailed *t*‐test. (D) mRNA expression of RNAPOL1 subunits (*POLR1A* and *POLR1B*) and components of RNAPOL1 preinitiation complex (*RRN3* and *UBTF*) in different CRC cell lines relative to the expression level in normal human colon mucosa analyzed via qPCR. Data show mean ± SD of technical replicates. The results are representative of 3 independent experiments with similar results obtained; unpaired, two‐tailed *t*‐test; *P* < 0.01 relative to normal human colon mucosa. (E) Analysis of the expression of RNAPOL1 subunits in data sets (Oncomine) from human CRCs (TT) relative to normal tissue (NT). Unpaired, two‐tailed t‐test. (F) 45S pre‐rRNA expression in different CRC cell lines relative to normal human colon mucosa analyzed via qPCR. The results are representative of 3 independent experiments with similar results obtained; unpaired, two‐tailed *t*‐test; *P* < 0.01, relative to normal human colon mucosa.

**Fig. S2.** CX5461 does not impair RNAPOL2 and RNAPOL3 function. (A) Immunoblot of LS174t cells treated with CX5461 (500 nm) or a control vehicle for 24 h of indicated proteins. The blot is representative of 2 independent experiments with similar results obtained. (B) Expression levels of 5S rRNA and 7SL RNA in LS174t treated with CX5461 (500 nm) or control vehicle (Ctr) for 24 h analyzed via qPCR (representative of 2 independent experiments with similar results obtained); unpaired, two‐tailed *t*‐test.

**Fig. S3.** CX5461 induces differentiation in CRC cells. (A) RNA‐Seq followed by GSEA of gene expression changes in murine Apc‐deleted vs. Apc WT mucosa. Heatmaps depicting regulation of genes of intestinal differentiation status in Apc‐deleted vs. WT situation. Calculation of the normalized enrichment score (NES) is based on a weighted running sum statistic and computed as part of the GSEA methodology. A Kolmogorov–Smirnov test with 1000 permutations was used to calculate *P* values that were then corrected for multiple testing using the Benjamini–Hochberg procedure (FDR). (B) GSEA enrichment plots comparing intestinal mucosa from WT mice to mucosa from mice with a bi‐allelic deletion of Apc. (C) mRNA expression of *KRT20* and *MUC2* in HCT116, DLD1, HT29, and SW480 cells treated with CX6461 (CX, 500 nm for 72 h) or a control vehicle (Ctr). Data show mean ± SD. Results are representative of 3 independent experiments with similar results obtained; unpaired, two‐tailed *t*‐test.

**Fig. S4.** CX5461 induces features of senescence in CRC cells. (A) mRNA expression and immunoblot of *CDKN1A*/p21 in LS174t cells treated with CX5461 (CX, 500 nm for 72 h) or a control vehicle (Ctr). Data show mean ± SD. Results are representative of 3 independent experiments with similar results obtained; unpaired, two‐tailed *t*‐test. (B) Immunoblot of LS174t cells treated with CX5461 (500 nM) or a control vehicle for 7 days for indicated proteins. The blot is representative of 3 independent experiments with similar results obtained. (C) Immunoblot of MTOs treated with CX5461 (500 nm) or a control vehicle for 7 days for indicated proteins. The blot is representative of 2 independent experiments with similar results obtained. (D) Staining of DLD1, HCT116, HT29, and SW480 cells for senescence‐associated β‐galactosidase activity after 7 days of treatment with CX5461 (500 nm) or a control vehicle. Images are representative of 2 independent experiments with similar results obtained; scale bar 200 μm. (E) Expression levels of 45S pre‐rRNA in LS174t treated with CX5461 (500 nm for indicated time) or control vehicle analyzed via qPCR. ‘ON’ samples are treated all the time with CX5461; ‘wash 72 h’ samples are treated with CX5461 for 72 h and further incubation for 72 h after inhibitor washout. Results are representative of 3 independent experiments with similar results obtained; unpaired, two‐tailed *t*‐test. (F) Quantification of O‐propargyl‐puromycin (OPP) incorporation to measure protein synthesis rate in LS174T cells treated with control vehicle (Ctr), CX5461 (CX, 500 nm for indicated time). Cells were incubated with 20 μm Click‐iT OPP for 30 min. Protein translation was blocked by cycloheximide (CHX, 178 nm) and added to the cells 15 min before OPP incubation. Shown is one experiment with technical replicates representative of 2 independent experiments with similar results. (G) RNA‐Seq of LS174t cells treated with CX5461 or control vehicle for 6, 24, and 72 h. Heatmap depicting regulated RNAPOL1 machinery‐associated genes.

**Fig. S5.** CX5461 induces RB signaling in CRC cells. (A) RNA‐Seq of LS174t cells treated with CX5461 or control vehicle for 6, 24, and 72 h. Heatmap depicting regulated gene sets (left). GSEA enrichment plots (right) of annotated gene sets for RB signaling in CX5461‐treated cells (72 h) in comparison with control samples. (B) Immunoblot of LS174t cells treated with CX5461 (500 nm for 7 days) or a control vehicle for indicated proteins. The blot is representative of 2 independent experiments with similar results obtained. (C) Expression levels of 45S pre‐rRNA in LS174t treated with CX5461 (500 nm for indicated time) or control vehicle analyzed via qPCR. ‘ON’ samples are treated all the time with CX5461; ‘wash 72 h’ samples are treated with CX5461 for 72 h and further incubation for 72 h after inhibitor washout. Results are representative of 3 independent experiments with similar results obtained; unpaired, two‐tailed *t*‐test. (E) Quantification of O‐propargyl‐puromycin (OPP) incorporation to estimate protein synthesis rate in LS174T cells treated with control vehicle (Ctr), CX5461 (CX, 500 nm for indicated time). Cells were incubated with 20 μm Click‐iT OPP for 30 min. Protein translation was blocked by cycloheximide (CHX, 178 nm) and added to the cells 15 min before OPP incubation. Shown is one experiment with technical replicates representative of 2 independent experiments with similar results (DLD1 and HCT116 10× magnification; HT29 and SW480 40× magnification). (F) RNA‐Seq of LS174t cells treated with CX5461 or control vehicle for 6, 24, and 72 h. Heatmap depicting regulated RNAPOL1 machinery‐associated genes.

**Fig. S6.** Sequential treatment of CRC cells with RNAPOLI inhibitor followed by a senolytic drug shows additive effects on cell viability. (A) Crystal violet staining of HCT116, DLD1, HT29, and SW480 cells treated with control vehicle, CX5461 (500 nm) or Navitoclax (500 nm) as single treatment or CX5461 and Navitoclax as sequential treatment for 7 days. Images are representative of 3 independent experiments with similar results obtained. (B) Crystal violet staining of HCT116, DLD1, HT29, and SW480 cells treated with control vehicle, CX5461 (500 nm) or ouabain (500 nm) as single treatment or CX5461 and ouabain as sequential treatment for 7 days. Images (2.5× magnification) are representative of 3 independent experiments with similar results obtained.

**Fig. S7.** CX5461 does not impair normal *in vivo* homeostasis. (A) Development of body weight of CX5461 and control vehicle‐treated mice described in Fig. 6A. Body weight was assessed daily for *n* = 4 mice per group. (B) Analysis of liver enzymes GOT and GPT in serum samples, and leucocytes, thrombocytes, red blood cells, and hemoglobin parameter (Hb) from mice described in Fig. 6A. Time point of taking blood samples was at the end of the experiment by cardiac puncture of *n* = 4 mice per group.

**Table S1.** shRNA sequences
**Table S2**. Antibodies used in immunofluorescence, immunohistochemistry, and immunoblotting
**Table S3**. Primers used for qPCR.

## Data Availability

All data are available from the authors on request. RNA‐Seq data are available from GEO IDs GSE204749 and GSE106858.
