# Supplementary material for: RNA polymerase I inhibition induces terminal differentiation, growth arrest, and vulnerability to senolytics in colorectal cancer cells
Source: Mol Oncol. 2022 Jul 1;16(15):2788–809. doi: 10.1002/1878-0261.13265 (PMC9348601; doi:10.1002/1878-0261.13265)

Supplementary Figure 1 Otto et al.

A

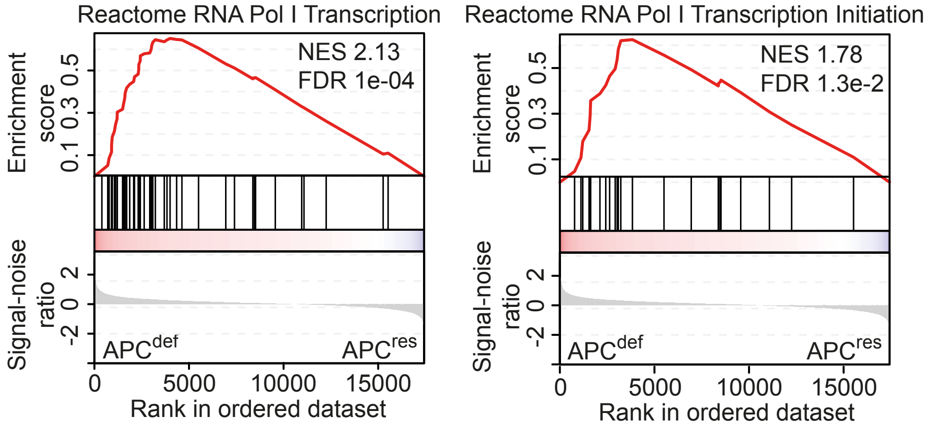

B

Gene Set	TCGA NES	TCGA FDR	Apc$^{fl/fl}$ NES	Apc$^{fl/fl}$ FDR	SW480 APC^{def} NES	SW480 APC^{def} FDR
Reactome RNA Polymerase I Transcription Initiation	1.43	0.433	2.11	0	-	-
Reactome RNA Polymerase I Promoter Escape	1.35	0.191	1.59	0.035	-	-
Reactome RNA Pol I Transcription Termination	1.17	0.211	-	-	1.82	0.009
GO Transcription Initiation from RNA Polymerase I Promoter	1.19	0.210	1.99	0	-	-
GO Transcription Elongation from RNA Polymerase I Promoter	1.20	0.297	2.04	0	-	-

C

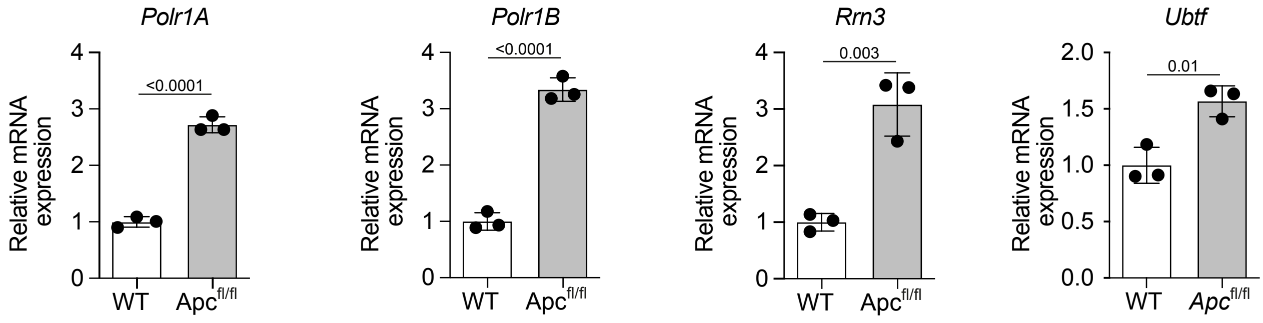

D

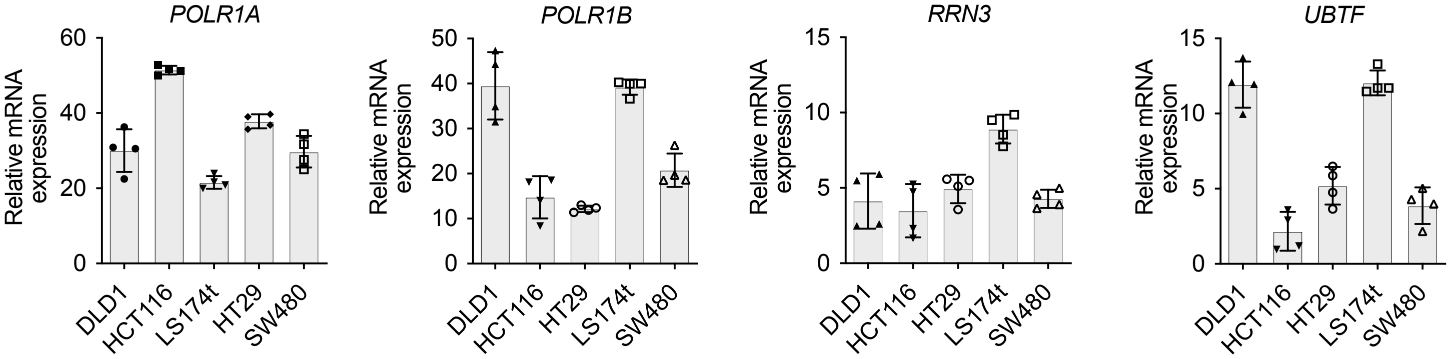

E

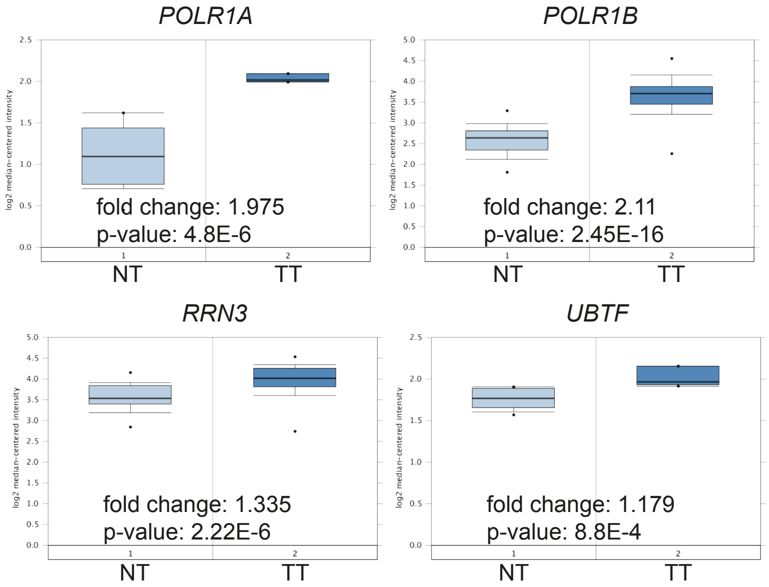

F

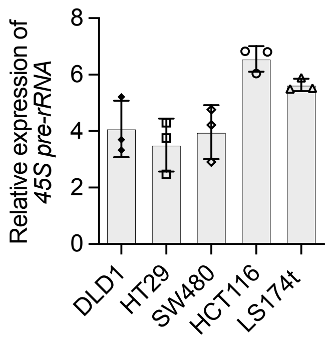

Supplement: Supplementary file 1 — Fig. S1. Oncogenic activation of WNT pathway upregulates RNAPOL1 machinery and its functional activity. (A) RNA‐Seq followed by GSEA of gene expression changes in APCdef and APCres cells (48 h ethanol and doxycycline treatment, respectively). Enrichment plots of indicated gene sets are displayed. Calculation of the normalized enrichment score (NES) is based on a weighted running sum statistic and computed as part of the GSEA methodology. A Kolmogorov–Smirnov test with 1000 permutations was used to calculate P values that were then corrected for multiple testing using the Benjamini–Hochberg procedure (FDR). (B) RNA‐Seq analysis comparing intestinal mucosa from WT mice to mucosa from mice with a bi‐allelic deletion of APC or APCdef and APCres cells (48 h ethanol and doxycycline treatment, respectively), and analysis from the TCGA database comparing human WT mucosa with CRC samples. (C) mRNA expression of RNAPOL1 subunits (Polr1a and Polr1b) and components of RNAPOL1 preinitiation complex (Rrn3 and Ubtf) in Apcfl/fl murine organoids relative to the expression level in WT organoids analyzed via qPCR. Data show mean ± SD of technical replicates. The results are representative of 3 independent experiments with similar results obtained; unpaired, two‐tailed t‐test. (D) mRNA expression of RNAPOL1 subunits (POLR1A and POLR1B) and components of RNAPOL1 preinitiation complex (RRN3 and UBTF) in different CRC cell lines relative to the expression level in normal human colon mucosa analyzed via qPCR. Data show mean ± SD of technical replicates. The results are representative of 3 independent experiments with similar results obtained; unpaired, two‐tailed t‐test; P < 0.01 relative to normal human colon mucosa. (E) Analysis of the expression of RNAPOL1 subunits in data sets (Oncomine) from human CRCs (TT) relative to normal tissue (NT). Unpaired, two‐tailed t‐test. (F) 45S pre‐rRNA expression in different CRC cell lines relative to normal human colon mucosa analyzed via qPCR. The r [file MOL2-16-2788-s004.pdf]
