# Supplementary figures and images for: RNA polymerase I inhibition induces terminal differentiation, growth arrest, and vulnerability to senolytics in colorectal cancer cells
Source: Mol Oncol. 2022 Jul 1;16(15):2788–809. doi: 10.1002/1878-0261.13265 (PMC9348601; doi:10.1002/1878-0261.13265)

A

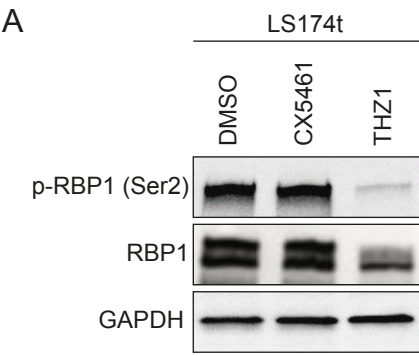

B

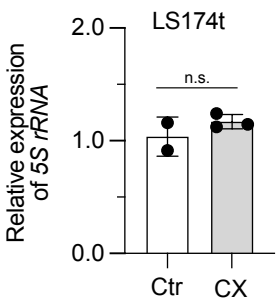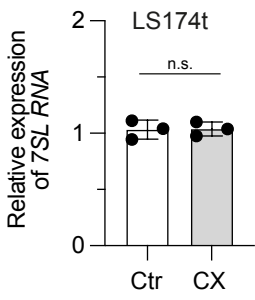

Supplement: Supplementary file 2 — Fig. S2. CX5461 does not impair RNAPOL2 and RNAPOL3 function. (A) Immunoblot of LS174t cells treated with CX5461 (500 nm) or a control vehicle for 24 h of indicated proteins. The blot is representative of 2 independent experiments with similar results obtained. (B) Expression levels of 5S rRNA and 7SL RNA in LS174t treated with CX5461 (500 nm) or control vehicle (Ctr) for 24 h analyzed via qPCR (representative of 2 independent experiments with similar results obtained); unpaired, two‐tailed t‐test. [file MOL2-16-2788-s005.pdf]

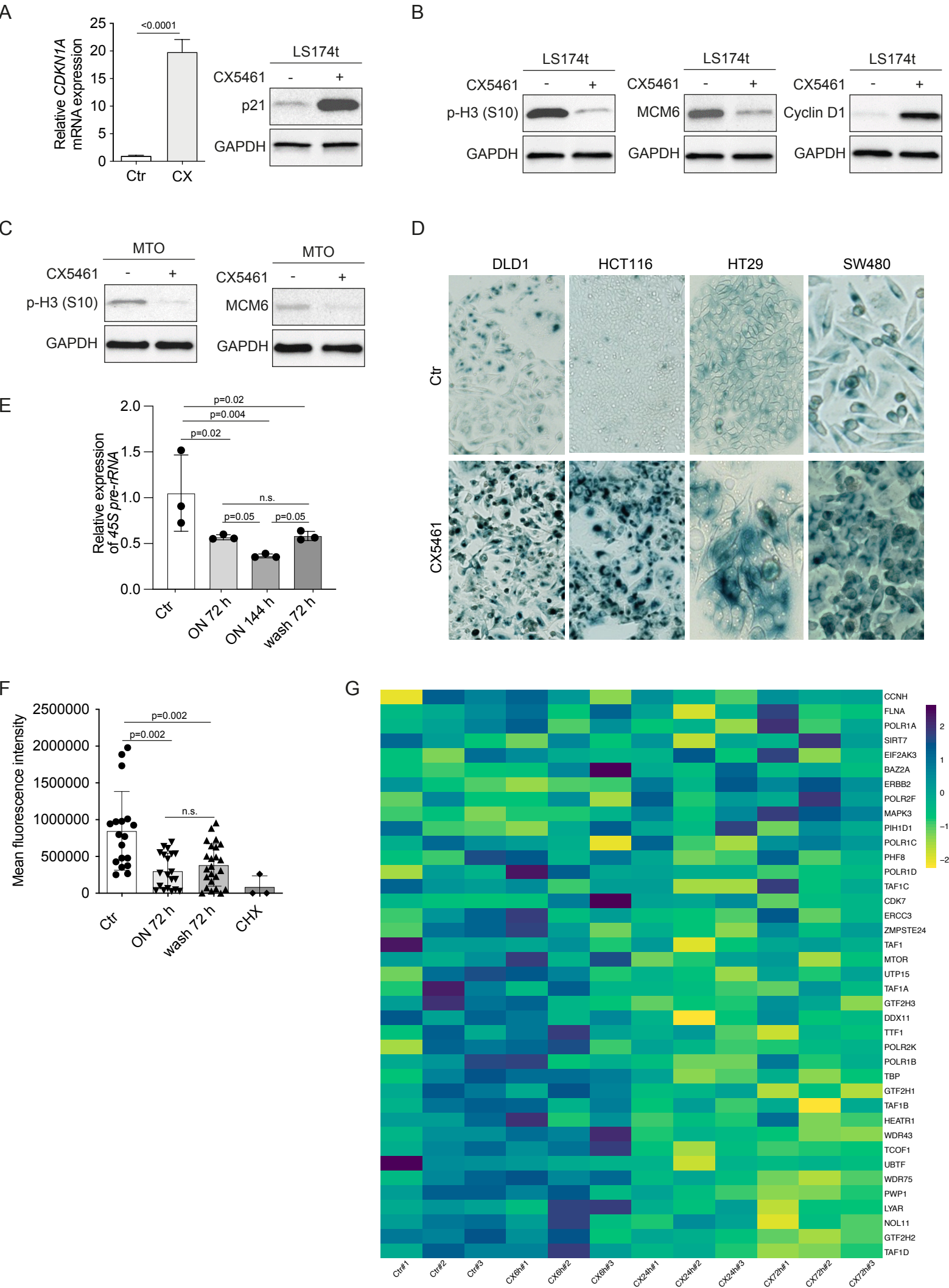

Supplement: Supplementary file 4 — Fig. S4. CX5461 induces features of senescence in CRC cells. (A) mRNA expression and immunoblot of CDKN1A/p21 in LS174t cells treated with CX5461 (CX, 500 nm for 72 h) or a control vehicle (Ctr). Data show mean ± SD. Results are representative of 3 independent experiments with similar results obtained; unpaired, two‐tailed t‐test. (B) Immunoblot of LS174t cells treated with CX5461 (500 nM) or a control vehicle for 7 days for indicated proteins. The blot is representative of 3 independent experiments with similar results obtained. (C) Immunoblot of MTOs treated with CX5461 (500 nm) or a control vehicle for 7 days for indicated proteins. The blot is representative of 2 independent experiments with similar results obtained. (D) Staining of DLD1, HCT116, HT29, and SW480 cells for senescence‐associated β‐galactosidase activity after 7 days of treatment with CX5461 (500 nm) or a control vehicle. Images are representative of 2 independent experiments with similar results obtained; scale bar 200 μm. (E) Expression levels of 45S pre‐rRNA in LS174t treated with CX5461 (500 nm for indicated time) or control vehicle analyzed via qPCR. ‘ON’ samples are treated all the time with CX5461; ‘wash 72 h’ samples are treated with CX5461 for 72 h and further incubation for 72 h after inhibitor washout. Results are representative of 3 independent experiments with similar results obtained; unpaired, two‐tailed t‐test. (F) Quantification of O‐propargyl‐puromycin (OPP) incorporation to measure protein synthesis rate in LS174T cells treated with control vehicle (Ctr), CX5461 (CX, 500 nm for indicated time). Cells were incubated with 20 μm Click‐iT OPP for 30 min. Protein translation was blocked by cycloheximide (CHX, 178 nm) and added to the cells 15 min before OPP incubation. Shown is one experiment with technical replicates representative of 2 independent experiments with similar results. (G) RNA‐Seq of LS174t cells treated with CX5461 or control vehicle for 6, 24, and 72 h. Heatmap depicti [file MOL2-16-2788-s008.pdf]

A

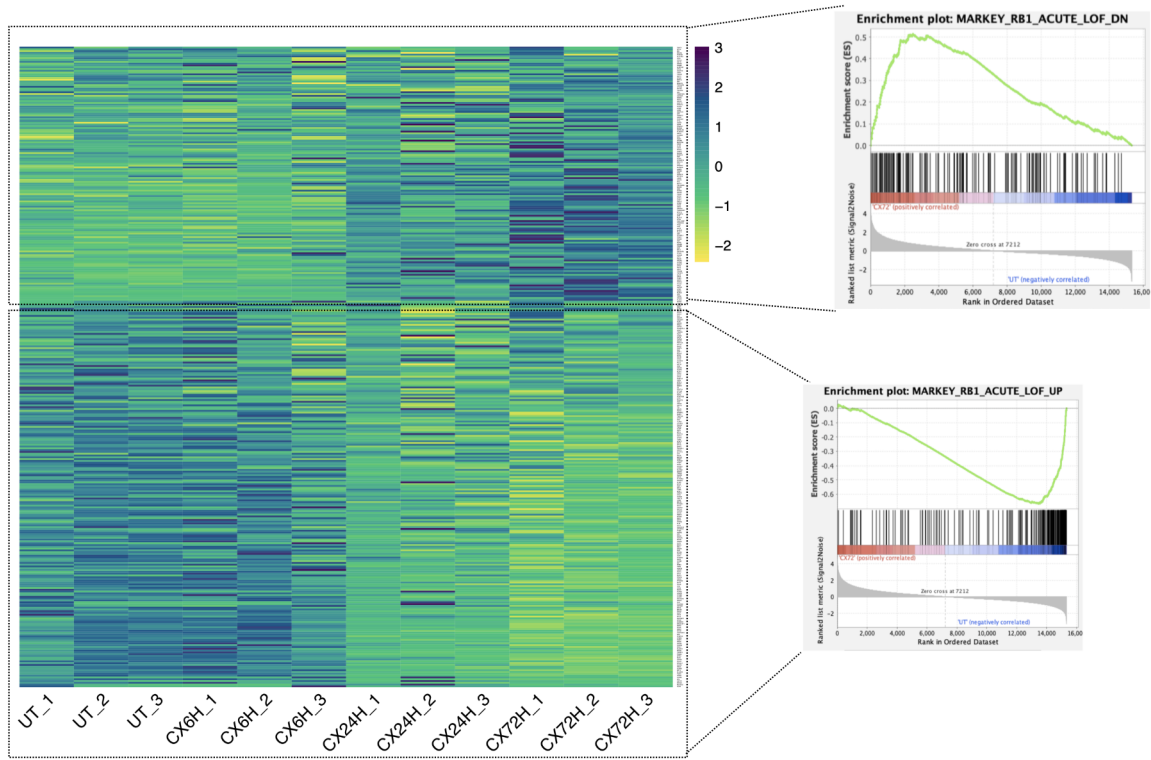

B

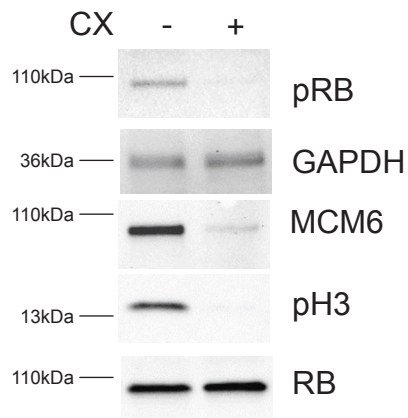

Supplement: Supplementary file 5 — Fig. S5. CX5461 induces RB signaling in CRC cells. (A) RNA‐Seq of LS174t cells treated with CX5461 or control vehicle for 6, 24, and 72 h. Heatmap depicting regulated gene sets (left). GSEA enrichment plots (right) of annotated gene sets for RB signaling in CX5461‐treated cells (72 h) in comparison with control samples. (B) Immunoblot of LS174t cells treated with CX5461 (500 nm for 7 days) or a control vehicle for indicated proteins. The blot is representative of 2 independent experiments with similar results obtained. (C) Expression levels of 45S pre‐rRNA in LS174t treated with CX5461 (500 nm for indicated time) or control vehicle analyzed via qPCR. ‘ON’ samples are treated all the time with CX5461; ‘wash 72 h’ samples are treated with CX5461 for 72 h and further incubation for 72 h after inhibitor washout. Results are representative of 3 independent experiments with similar results obtained; unpaired, two‐tailed t‐test. (E) Quantification of O‐propargyl‐puromycin (OPP) incorporation to estimate protein synthesis rate in LS174T cells treated with control vehicle (Ctr), CX5461 (CX, 500 nm for indicated time). Cells were incubated with 20 μm Click‐iT OPP for 30 min. Protein translation was blocked by cycloheximide (CHX, 178 nm) and added to the cells 15 min before OPP incubation. Shown is one experiment with technical replicates representative of 2 independent experiments with similar results (DLD1 and HCT116 10× magnification; HT29 and SW480 40× magnification). (F) RNA‐Seq of LS174t cells treated with CX5461 or control vehicle for 6, 24, and 72 h. Heatmap depicting regulated RNAPOL1 machinery‐associated genes. [file MOL2-16-2788-s007.pdf]

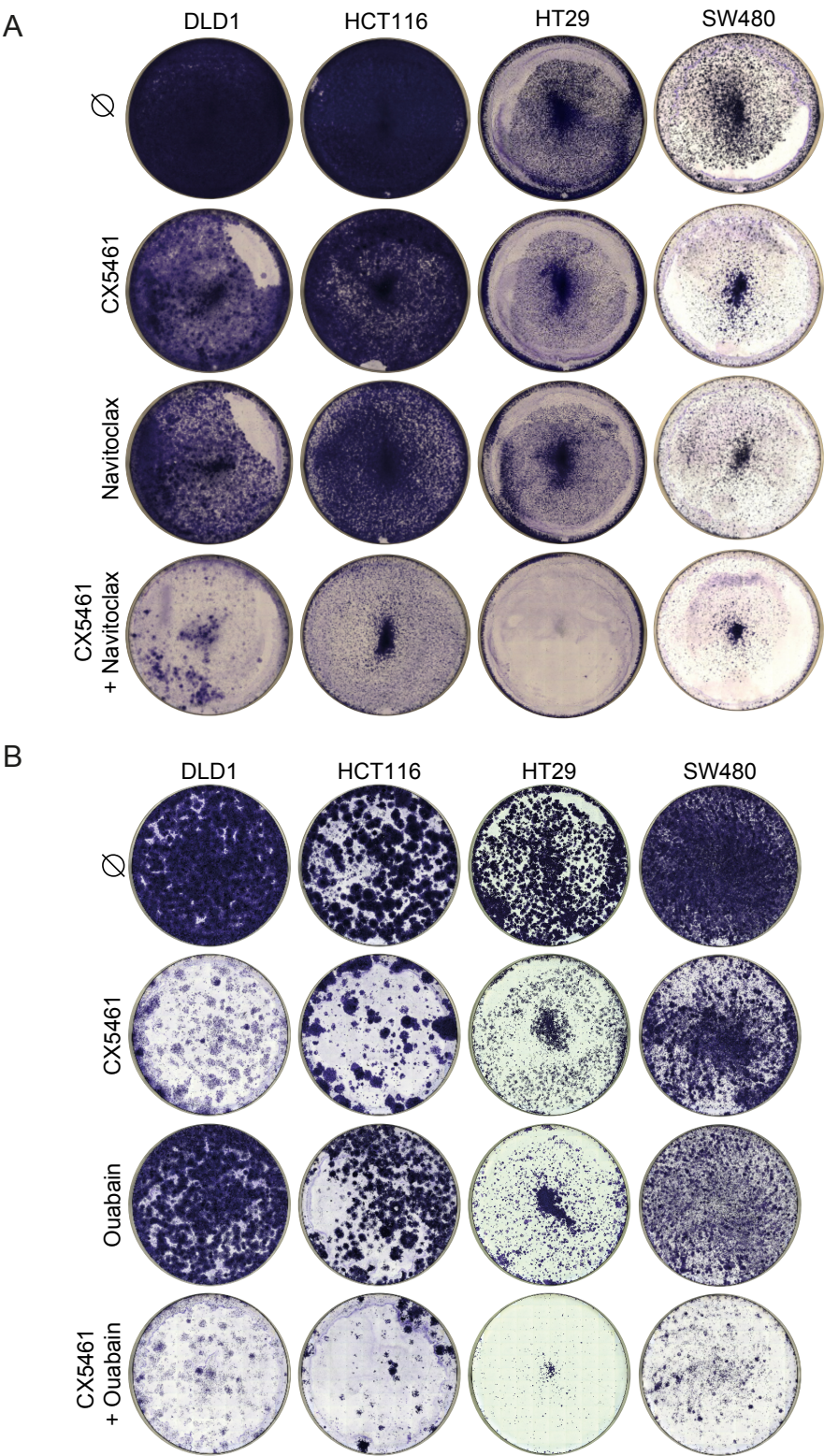

Supplement: Supplementary file 6 — Fig. S6. Sequential treatment of CRC cells with RNAPOLI inhibitor followed by a senolytic drug shows additive effects on cell viability. (A) Crystal violet staining of HCT116, DLD1, HT29, and SW480 cells treated with control vehicle, CX5461 (500 nm) or Navitoclax (500 nm) as single treatment or CX5461 and Navitoclax as sequential treatment for 7 days. Images are representative of 3 independent experiments with similar results obtained. (B) Crystal violet staining of HCT116, DLD1, HT29, and SW480 cells treated with control vehicle, CX5461 (500 nm) or ouabain (500 nm) as single treatment or CX5461 and ouabain as sequential treatment for 7 days. Images (2.5× magnification) are representative of 3 independent experiments with similar results obtained. [file MOL2-16-2788-s006.pdf]

A

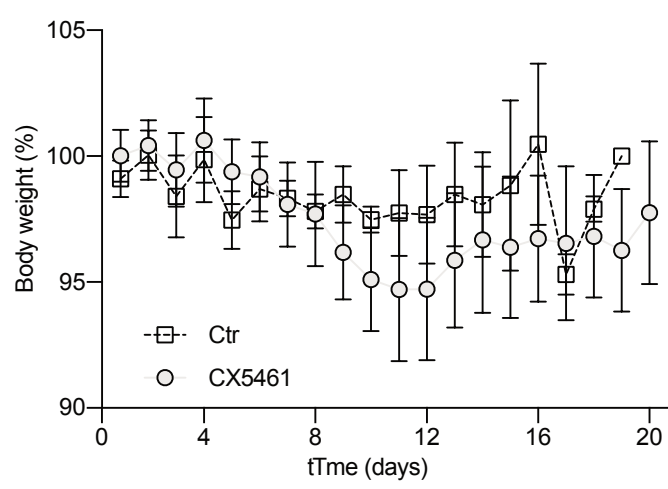

B

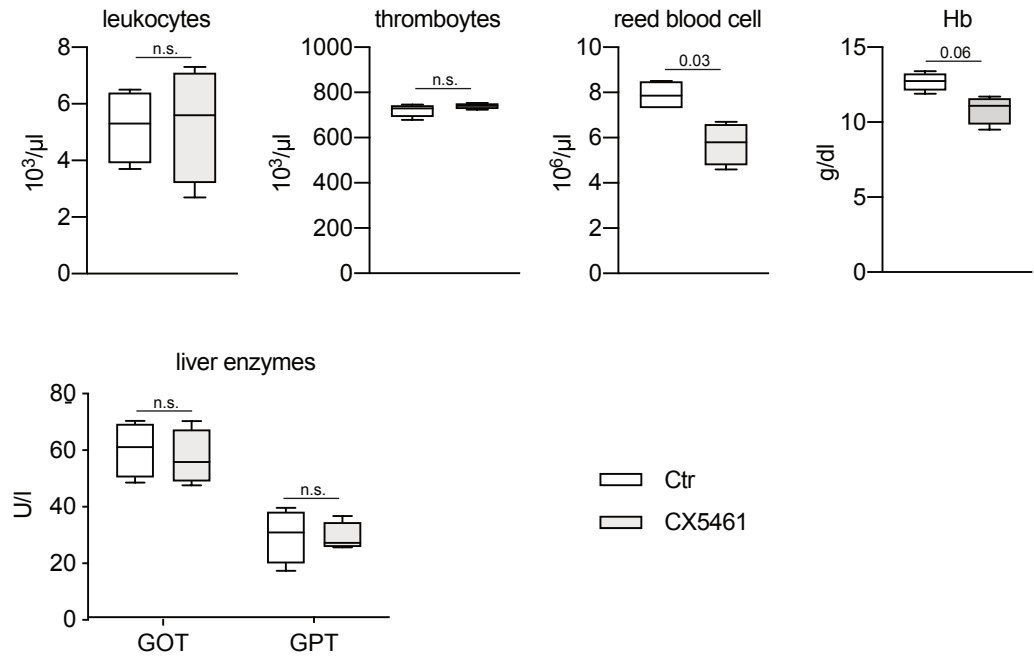

Supplement: Supplementary file 7 — Fig. S7. CX5461 does not impair normal in vivo homeostasis. (A) Development of body weight of CX5461 and control vehicle‐treated mice described in Fig. 6A. Body weight was assessed daily for n = 4 mice per group. (B) Analysis of liver enzymes GOT and GPT in serum samples, and leucocytes, thrombocytes, red blood cells, and hemoglobin parameter (Hb) from mice described in Fig. 6A. Time point of taking blood samples was at the end of the experiment by cardiac puncture of n = 4 mice per group. [file MOL2-16-2788-s001.pdf]
