# Supplementary material for: RNA polymerase I inhibition induces terminal differentiation, growth arrest, and vulnerability to senolytics in colorectal cancer cells
Source: Mol Oncol. 2022 Jul 1;16(15):2788–809. doi: 10.1002/1878-0261.13265 (PMC9348601; doi:10.1002/1878-0261.13265)

Supplementary Figure 3

Otto et al.

A

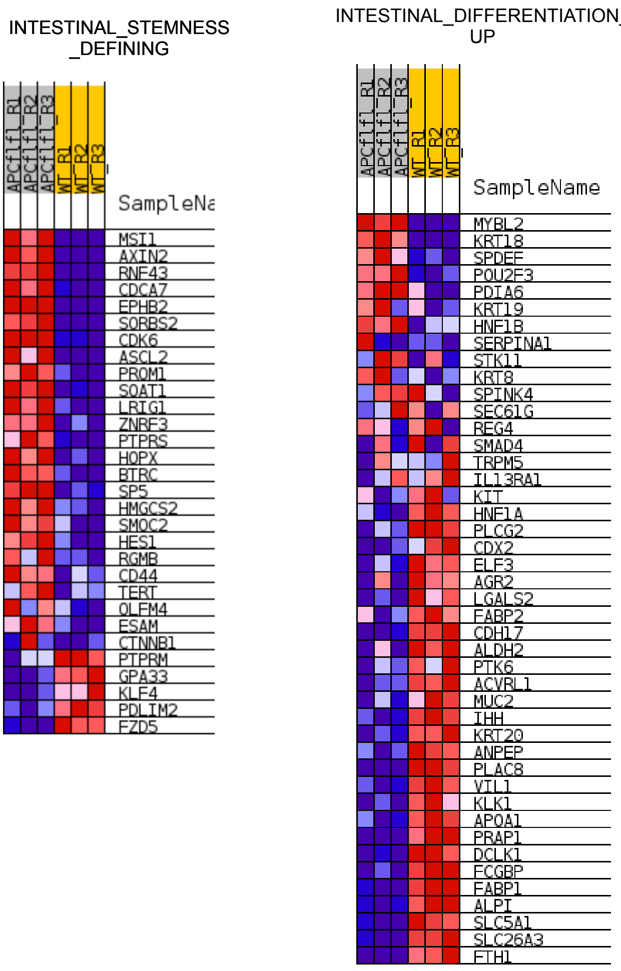

B

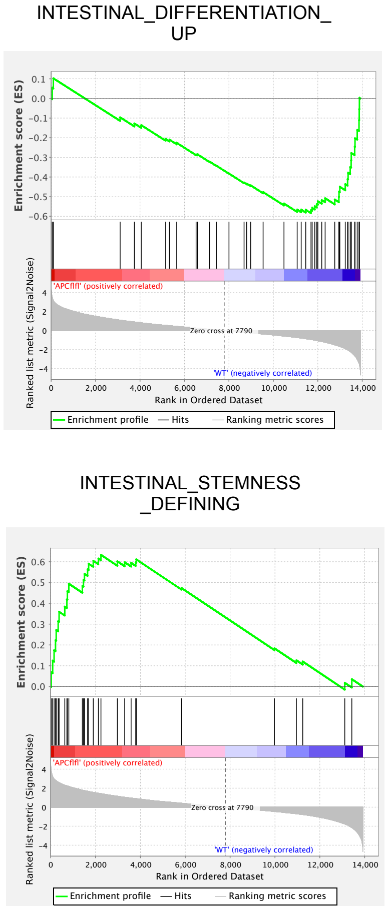

C

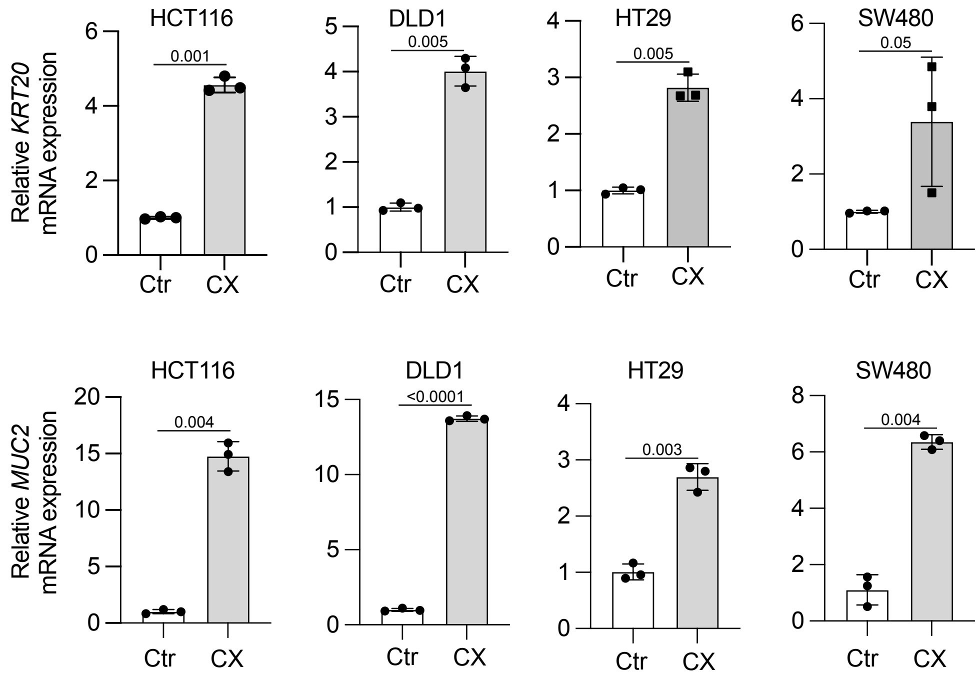

Supplement: Supplementary file 3 — Fig. S3. CX5461 induces differentiation in CRC cells. (A) RNA‐Seq followed by GSEA of gene expression changes in murine Apc‐deleted vs. Apc WT mucosa. Heatmaps depicting regulation of genes of intestinal differentiation status in Apc‐deleted vs. WT situation. Calculation of the normalized enrichment score (NES) is based on a weighted running sum statistic and computed as part of the GSEA methodology. A Kolmogorov–Smirnov test with 1000 permutations was used to calculate P values that were then corrected for multiple testing using the Benjamini–Hochberg procedure (FDR). (B) GSEA enrichment plots comparing intestinal mucosa from WT mice to mucosa from mice with a bi‐allelic deletion of Apc. (C) mRNA expression of KRT20 and MUC2 in HCT116, DLD1, HT29, and SW480 cells treated with CX6461 (CX, 500 nm for 72 h) or a control vehicle (Ctr). Data show mean ± SD. Results are representative of 3 independent experiments with similar results obtained; unpaired, two‐tailed t‐test. [file MOL2-16-2788-s002.pdf]
