# Supplementary material for: RNA polymerase I inhibition induces terminal differentiation, growth arrest, and vulnerability to senolytics in colorectal cancer cells
Source: Mol Oncol. 2022 Jul 1;16(15):2788–809. doi: 10.1002/1878-0261.13265 (PMC9348601; doi:10.1002/1878-0261.13265)
Supplement: Supplementary file 8 — Table S1. shRNA sequences Table S2. Antibodies used in immunofluorescence, immunohistochemistry, and immunoblotting Table S3. Primers used for qPCR. [file MOL2-16-2788-s003.docx]

| pGIPZ or pInducer10 shMiz1 #1 | TGCTGTTGACAGTGAGCGAACACTGCGACAAGAAGTTCAATAGTGAAGCCACAGATGTATTGAACTTCTTGTCGCAGTGTGTGCCTACTGCCTCGGA |
| --- | --- |
| pGIPZ or pInducer10 shMiz1 #5 | TGCTGTTGACAGTGAGCGAGCCCTTCTGACTGTTTATTTATAGTGAAGCCACAGATGTATAAATAAACAGTCAGAAGGGCCTGCCTACTGCCTCGGA |

**Supp. Tab. 1shRNA sequences**

|  | *company / catalog no.* | *dilution* | *used for* |
| --- | --- | --- | --- |
| Rabbit anti-Krt20 | Cell Signaling Technology / 13063 | 1:100  1:500  1:1000 | Immunofluorescence  organoids  cells  immunoblot |
| Rabbit anti-Ki67 | Abcam / Ab15580 | 1:500 | immunohistochemistry |
| Mouse anti-NPM1 | Abcam / AB10530 | 1:500 | immunofluorescence |
| Rabbit anti-POLR1A | Sigma-Aldrich / HPA031513 | 1:200 | immunohistochemistry |
| Rabbit anti-RPL29 | GeneTex / GTX101833 | 1:500 | immunofluorescence |
| Rabbit anti-UBTF | Sigma-Aldrich / HPA006385 | 1:200 | immunohistochemistry |
| Mouse anti-VCL | Sigma-Aldrich / V9131 | 1:2000 | immunoblot |
| ECL-anti-rabbit IgG horseradish  Peroxidase | GE Healthcare / Fisher  Scientific GmbH / 1079-4347 | 1:15000 | immunoblot |
| ECL-anti-mouse IgG horseradish  peroxidase | GE Healthcare / Fisher  Scientific GmbH / 1019-6124 | 1:15000 | immunoblot |
| Anti rabbit immunoglobulins/HRP | DAKO / P 0448 | 1:200 | immunohistochemistry |
| Anti rabbit IgG Alexa-Fluor | Cell Signaling Technology | 1:2000 | immunofluorescence |
| Goat anti-mouse IgG (H+L) highly cross-  adsorbed secondary antibody, Alexa  Fluor 488 | Thermo Fisher Scientific / A-11029 | 1:400 | immunofluorescence |
| Goat anti-rabbit IgG (H+L) highly cross-  adsorbed secondary antibody, Alexa  Fluor 488 | Thermo Fisher Scientific / A-11034 | 1:400 | immunofluorescence |
| Mouse Anti Human α-Tubulin | Sigma-Aldrich | T6074 | immunoblot |
| Mouse Anti Human GAPDH (GAPDH-71.1) | Sigma-Aldrich | G9295 | immunoblot |
| Mouse Anti Human Lamin A (133A2) | Cell Signaling Technology | 86846 | immunoblot |
| Rabbit Anti Human Cyclin D1 | Cell Signaling Technology | 2922 | immunoblot |
| Rabbit Anti Human MCM6 | Bethyl Laboratories | A300-194A-M | immunoblot |
| Rabbit Anti Human Phospho-Histone H3 (Ser10) | Invitrogen | 44-1190G | immunoblot |
| Rabbit Anti Human Phospho-Rb (Ser795) | Cell Signaling Technology | 9301 | immunoblot |
| Rabbit Anti Human Phospho-Rpb1 CTD (Ser2) | Cell Signaling Technology | 3499 | immunoblot |
| Rabbit Anti Human RPL23 | Proteintech | 16086-1-AP | immunoblot |
| Rabbit Anti Human RPL29 | GeneTex | GTX101833 | immunofluorescence |
| Rabbit Anti Human RPS14 | Bethyl Laboratories | A304-031A-T | immunoblot |
| p21 Waf1/Cip1 (12D1) | Cell Signaling Technology | 2947 | immunoblot |
| Mouse Anti Human POL II (Rpb1) | Santa Cruz | sc-55492 | immunoblot |

**Supp. Tab. 2 Antibodies used in immunofluorescence, immunohistochemistry and for immunoblotting**

|  | *forward* | *reverse* |
| --- | --- | --- |
| **ACTB** | cctcgcctttgccgatcc | ggatcttcatgaggtagtcagtc |
| **B2M** | gtgctcgcgctactctctc | gtcaacttcaatgtcggat |
| **CDKN1A/P21** | cgatgccaacctcctcaacga | tcgcagacctccagcatcca |
| **GAPDH** | TCTCCTCTGACTTCAACAGCGAC | CCCTGTTGCTGTAGCCAAATTC |
| **KRT20 I** | aactgcaaaatgctcggtgt | caggccttggagatcagctt |
| **KRT20 II** | AACTAACGGAGCTGAGACGC | TGGTCTCCTCTAGAGTGTGCT |
| **LGR5** | cagcgtcttcacctcctacc | tccaggaagcggagactg |
| **MUC2** | caagatcttcatggggagga | gaacacggtggtcctcttgt |
| **POLR1A** | TCCTTCGGGATGTATTCGGC | CAGGCTGTCCAGGTATCGAG |
| **POLR1B** | CCTCAACACCGGGGAAAGTT | CACTCCCAGTGTCATTACTGC |
| **PPIA** | ggcaaatgctggacccaacaca | tgctggtcttgccattcct |
| **PPIA II** | tcctggcatcttgtccat | tgctggtcttgccattcct |
| **PPIA III** | cctaaagcatacgggtcctg | cactttgccaaacaccacat |
| **RPL0** | GCAATGTTGCCAGTGTCTG | GCCTTGACCTTTTCAGCAA |
| **RRN3** | GAAGAACCAGCTGTTAGATCCAGA | AGAACGGAATTCTAGCAGCCA |
| **45 S pre-rRNA I** | CTCGCCAAATCGACCTCGTA | GCTAGCTGCGTTCTTCATCG |
| **45 S pre-rRNA II** | CCGCGCTCTACCTTACCTACCT | GCATGGCTTAATCTTTGAGACAAG |
| **45 S pre-rRNA III** | ggttcgaggcggtttgagt | gacgtgcgctcaccgag |
| **47 S pre-rRNA** | cgacctgtcgtcggagag | ggacgcgcgagagaacag |
| **UBTF** | GCACCTGGACCTCTGGGTTA | CGCAGCTTGGTCATGCTCTT |
| **ZBTB17/MIZ1** | GACCCCACTTCCAAGATGC | GGCCTTCAGGTTCCCTACC |
| **5S rRNA** | GGCCATACCACCCTGAACGC | CAGCACCCGGTATTCCCAGG |
| **7SL I** | GGGCTGTAGTGCGCTATGC | CCCGGGAGGTCACCATATT |
| **7SL II** | GTGTCCGCACTAAGTTCGGCATCAATATGG | TATTCACAGGCGCGATCCCACTACTGATC |
| **RPL0** | GCAATGTTGCCAGTGTCTG | GCCTTGACCTTTTCAGCAA |

**Supp. Tab. 3 Primers used for qPCR**
